# Aphid effector pair Mp1–Mp58 forms an effector complex that targets a host trafficking protein

**DOI:** 10.1093/jxb/erag070

**Published:** 2026-02-21

**Authors:** Jade R Bleau, Namami Gaur, S Ronan Fisher, Thomas Waksman, Michael Porter, Jorunn I B Bos

**Affiliations:** Division of Plant Sciences, Faculty of Life Sciences, University of Dundee, Dundee DD2 5DA, UK; Division of Plant Sciences, Faculty of Life Sciences, University of Dundee, Dundee DD2 5DA, UK; Division of Plant Sciences, Faculty of Life Sciences, University of Dundee, Dundee DD2 5DA, UK; Division of Plant Sciences, Faculty of Life Sciences, University of Dundee, Dundee DD2 5DA, UK; Cell and Molecular Sciences, The James Hutton Institute, Invergowrie, Dundee DD2 5DA, UK; Division of Plant Sciences, Faculty of Life Sciences, University of Dundee, Dundee DD2 5DA, UK; Cell and Molecular Sciences, The James Hutton Institute, Invergowrie, Dundee DD2 5DA, UK; Universitat Jaume I, Spain

**Keywords:** Effectors, host trafficking, insect–herbivore, molecular dialogue, *Myzus persicae*, protein complex

## Abstract

Pathogen and pest effectors play a crucial role in manipulating plant biological processes, facilitating infection and infestation. While pathogens and pests secrete repertoires of effectors into host plants, most effector function studies focus on characterizing individual proteins. Our previous work identified a genetically linked and co-regulated gene pair in the aphid pest *Myzus persicae* encoding effectors Mp1 and Mp58. Here, we explored the functional link between these two effectors. We revealed that effectors Mp1 and Mp58 interact *in planta* and *in vitro* and form an oligomeric complex. The putative orthologues of the Mp1–Mp58 pair in the aphid species *Rhopalosiphum padi*, Rp1 and Rp58, also interact, but members of the pair cannot interact across aphid species, suggesting that effector pairs have co-evolved within each aphid species but diversified across species. Both Mp1 and Mp58 associate with the host Vacuolar Protein Sorting associated Protein 52 (VPS52) to form an Mp1–Mp58–VPS52 complex, which localizes at vesicle-like structures. Our findings point to effector complex formation in plant–insect interactions and highlight a further layer of complexity in the molecular dialogue between insects and their host plants. Our work highlights the importance of considering the context in which effectors may function within a larger effector repertoire.

## Teaser

A highly conserved genetically linked and co-regulated effector pair in aphids forms an effector protein complex that targets a host trafficking protein.

## Introduction

Phloem-feeding insects, such as plant hoppers, leaf hoppers, whiteflies, and aphids, deliver molecules inside their host, known as effectors, to promote susceptibility. Effector delivery takes place during phloem-feeding and probing and involves the secretion of saliva via highly specialized mouthparts called stylets. Saliva containing effectors is secreted into the apoplast, phloem, and, depending on insect species, may also be delivered into cells along the stylet pathway (Naalden *et al.*, 2021; Wang *et al.*, 2023). Like plant pathogen effectors, which have extensively been studied over the past decades, phloem-feeding insect effectors target host proteins to modify their activity to promote host susceptibility, extending the effector paradigm to herbivorous insects. Advances in genomics and proteomics approaches have indeed unveiled effector repertoires of many herbivorous insect species, and characterization of their virulence activities remains a major challenge. With limited genetic crop resistance available against insect pests, understanding how effectors function and interfere with plant host cell biology promises to underpin the design of novel strategies for crop protection.

The aphid *Myzus persicae* (green peach aphid) is a major agricultural pest in part due to its exceptionally broad host range and ability to vector many important plant viruses (Blackman and Eastop, 2000). The *M. persicae* effector repertoire consists of diverse proteins, as predicted by saliva proteomics and bioinformatics pipelines (Harmel *et al.*, 2008; Bos *et al.*, 2010; Vandermoten *et al.*, 2014; Thorpe *et al.*, 2016, 2018), and features an over-representation of disordered proteins (Waksman *et al.*, 2024). Functional characterization of *M. persicae* effector proteins led to the identification of several host targets (Rodriguez *et al.*, 2017; Wang *et al.*, 2021; Liu *et al.*, 2022; Liu *et al*., 2025; Gravino *et al.*, 2025). The *M. persicae* effector Mp1 is expressed in salivary glands, and secreted in saliva as shown by proteomics (Harmel *et al.*, 2008; Bos *et al.*, 2010; Thorpe *et al.*, 2016). We previously showed that Mp1 can associate with Vacuolar Protein Sorting associated Protein 52 (VPS52) in a species-specific manner. Specifically, Mp1 was only able to interact with VPS52 proteins from host plant species *Solanum tuberosum* (StVPS52) and Arabidopsis (AtVPS52), but not with those from non-/poor host plants *Medicago truncatula* (MtVPS52) and *Hordeum vulgare* (HvVPS52). In line with these observations, Mp1 variants from aphid species unable to infest *S. tuberosum* and Arabidopsis did not interact with VPS52 from these plant species (Rodriguez *et al.*, 2017). Overexpression of StVPS52 reduced host susceptibility to aphids, and infestation led to a reduction of detectable VPS52 protein levels, indicating that VPS52 is an important virulence target. Targeting of host cellular trafficking machinery by plant parasites is a common feature in plant–pathogen interactions (Yuen *et al.*, 2023) and the association of aphid effector Mp1 with VPS52, a component of the Golgi Associated Retrograde Protein (GARP) complex, and degradation of VPS52 during infestation indicate this feature can be extended to plant–herbivorous insect interactions.

The *Mp1* effector gene is co-located with another effector gene, *Mp58*, within the *M. persicae* genome, and the expression of the Mp1–Mp58 effector gene pair is tightly co-regulated (Thorpe *et al.*, 2018). The effector genes are positioned in a head to tail orientation, around 5.5 kb apart, and feature a highly similar 5′ end promoter region. Moreover, the co-location of this gene pair is conserved across at least five aphid genomes, including the *Rhopalosiphum padi* genome, but with a lack of synteny in the corresponding genomic regions (Thorpe *et al*., 2018). Aphid gene expression analyses revealed tight co-regulation of Mp1(-like) and Mp58(-like) in different species as well as co-regulation with a set of putative effectors, pointing to a shared transcriptional control mechanism (Thorpe *et al.*, 2018).

The effector Mp58, like Mp1, can be detected in saliva by proteomics and is highly expressed in *M. persicae* salivary glands (Harmel *et al.*, 2008; Thorpe *et al.*, 2016). Ectopic expression of this effector in Arabidopsis, *Nicotiana tabacum*, or *Nicotiana benthamiana* leads to reduced susceptibility to *M. persicae*, suggesting this effector may trigger plant defence responses (Elzinga *et al*., 2014; Escudero-Martinez *et al.*, 2020). In contrast, the Mp58-like effector from *Macrosiphum euphorbiae*, called Me10, enhances tomato and *N. benthamiana* susceptibility to *M. euphorbiae* and *M. persicae* (Atamian *et al.*, 2013), and interacts with tomato 14-3-3 isoform 7 (TFT7), which contributes to defence against aphids (Chaudhary *et al.*, 2019). Ectopic expression of the putative orthologs Rp1 and Rp58 from *R. padi* in transgenic barley lines showed that Rp1, but not Rp58, promotes host susceptibility (Escudero-Martinez *et al*., 2020).

The co-location and co-regulation of the highly conserved *Mp1–Mp58* gene pair across aphid species point to a functional link between the encoded effector proteins. In this study we sought to explore whether there is indeed such a functional link between Mp1 and Mp58 by investigating physical protein–protein interaction, subcellular co-localization, and impact of the effector combination on aphid performance. We show that Mp1 and Mp58 physically interact to form an effector complex, and that both effectors can associate with VPS52, a previously identified Mp1 host target implicated in plant–aphid interactions. Computational modelling suggested that Mp1 and Mp58 associate with different regions of VPS52. We experimentally confirmed the interaction of Mp1 with the VPS52 N-terminus *in vitro* and *in planta* using host/nonhost VPS52 chimeras. Moreover, in co-expression assays with individual effector proteins, Mp1 relocalizes to VPS52-associated vesicles, while Mp58 remains cytoplasmic. However, when both effectors are co-expressed with VPS52, Mp58 can be detected together with Mp1 and VPS52 in vesicle-like structures pointing to the presence of an Mp58–Mp1–VPS52 protein complex. Our data highlight an unexplored layer of complexity in the plant–herbivorous insect molecular dialogue based on effector complex formation not only with host proteins but also with other effectors to potentially mediate and/or regulate virulence activities. Our study also highlights the importance of considering effector functions not in isolation, but in the context of secreted effector repertoires, with some effectors potentially mediating and/or regulating activities via interaction with other effectors.

## Materials and methods

### Plant growth conditions and aphid maintenance

*Nicotiana benthamiana* plants were grown in a glasshouse with 16 h of light at ∼25 °C.

*Myzus persicae* (Clone O) was maintained on *N. benthamiana* leaves in a growth cabinet with 16 h light (100–200 µmol m^−2^ s^−1^), 22 °C during the day, 20 °C at night, 65% relative humidity.

### Plasmids

Constructs for *in planta* co-immunoprecipitation (co-IP) experiments and confocal assays have been cloned and described previously: green fluorescent protein (GFP)–Mp1 (pB7WGF2), red fluorescent protein (RFP)–AtVPS52, RFP–HvVPS52 (pK7WGR2) (Rodriguez *et al.*, 2017), GFP–Mp58 (pB7WGF2) (Escudero-Martinez *et al.*, 2020), and myc–Mp1 and myc–Rp1 (pGWB21) (Rodriguez *et al.*, 2017). AtVPS52^1–170^, AtVPS52^Hv1–165^, and HvVPS52^At1–170^ were synthesized with the addition of AttL sites for Gateway cloning into the plasmid pTwist ENTR by Twist Bioscience. To generate RFP-tagged constructs for plant expression, AtVPS52^1–170^, AtVPS52^Hv1–165^, and HvVPS52^At1–170^ were cloned into pK7WGR2 by LR reaction (LR clonase II, Thermo Fisher Scientific, cat. no. 11791020) following the manufacturer’s instructions and transformed into *Escherichia coli* Top10 cells. Constructs were verified by sequencing and transformed in *Agrobacterium tumefaciens* (GV3101) for expression in *N. benthamiana*. To generate blue fluorescent protein (BFP2)–AtVPS52, previously cloned AtVPS52 in pEntr1A was cloned into pJRA109 (Allen *et al.*, 2025) by LR reaction according to the manufacturer’s instructions and transformed into *E. coli* Top10 cells. Constructs were verified by sequencing and transformed in *Agrobacterium tumefaciens* (GV3101) for expression in *N. benthamiana*.

For aphid assays pAtSUC2::Mp1 (pHW59) had be cloned previously (Rodriguez *et al.*, 2017). Mp58 was cloned from pEntr1A into pHW59 (Gottwald *et al.*, 2000) by LR reaction to generate pAtSUC2::Mp58. Constructs were verified by sequencing and transformed into *Agrobacterium* (GV3101) for expression in *N. benthamiana*.

For *E. coli* protein expression and purification, codon-optimized glutathione *S*-transferase (GST), AtVPS52, Mp1, and Mp58 coding DNA sequences were synthesized by Integrated DNA Technologies or Twist Bioscience. Regions of interest were PCR-amplified and assembled into a pET-15b expression vector using GeneArt Gibson Assembly HiFi Master Mix (Thermo Fisher Scientific). 6×His tags and TEV or HRV 3C protease cleavage sites were encoded in PCR primers.

### *Agrobacterium tumefaciens* infiltration for transient expression

*Agrobacterium tumefaciens* (strain GV3101) carrying the indicated constructs was grown shaking overnight at 28 °C. Cultures were centrifuged at 2500 *g* for 10 min at room temperature. Pellets were resuspended in infiltration buffer (10 mM MgCl_2_ and 10 mM MES, pH 5.6), the OD was adjusted (see below) and 200 µM acetosyringone was added to the infiltration buffer. For co-IP experiments *Agrobacterium* was diluted to OD_600_ 0.3 for all but GFP (OD_600_ 0.05) along with the silencing suppressor p19 (OD_600_ 0.1). For aphid performance assays the OD_600_ was 0.2 along with the silencing suppressor p19 (OD_600_ 0.1). For confocal-based co-localization experiments, the OD_600_ ranged from 0.1 to 0.2 for all, along with the silencing suppressor p19 (OD_600_ 0.1 for Mp1–Mp58 co-localization and 0.001 for all other experiments). Cultures were incubated for 2 h in the dark at 28 °C. Three- to four-week-old *N. benthamiana* plants were infiltrated with *Agrobacterium* carrying the indicated constructs.

### Co-immunoprecipitation

Leaf tissue from *N. benthamiana* was ground in liquid nitrogen and protein was extracted with GTEN buffer (10% glycerol, 25 mM Tris–HCl pH 7.5, 1 mM EDTA) containing 0.2% Triton X-100, 10 mM dithiothreitol (DTT) and 1× EDTA-free protease inhibitor cocktail (Thermo Fisher Scientific, cat. no. A32965). Samples were incubated for 10 min with frequent vortexing. Samples were centrifuged at 16 000*×g* for 20 min at 4 °C. The supernatant was diluted with IP buffer (GTEN buffer, 0.1% Tween-20) to reduce the DTT concentration to <10 mM. Lysates were incubated with either Chromtek myc-trap (Proteintech; ymta) or RFP-trap (Proteintech; rtma) rotating at 4 °C for 2 h. Beads were washed with IP buffer and protein was eluted at 80 °C for 10 min in 2× SDS sample buffer containing 50 mM DTT. Samples were stored at −20 °C until use.

### *Escherichia coli* protein expression and purification

The pET-15b expression vector encoding protein of interest was transformed into *E. coli* BL21 (DE3) cells. Overnight culture was inoculated into 1 litre lysogeny broth (LB) medium in a 2 litre Erlenmeyer flask, and cell culture was grown at 37 °C until OD_600_ 0.4–0.8 was reached. The culture temperature was reduced to 25 °C, and protein expression induced via addition of isopropyl β-D-1-thiogalactopyranoside (IPTG) (final concentration 0.1 mM). After incubation at 25 °C for 4 h, cells were harvested via centrifugation at 3500 *g* for 5 min, resuspended in ice-cold lysis buffer (25 mM HEPES, 150 mM NaCl, 25 mM imidazole, 1 mM DTT, pH 7.5) and stored at −20 °C. Thawed cell suspensions were lysed via sonication and subjected to centrifugation at 20 000 g for 30 min, and supernatant passed through a 0.45 µm syringe filter. Proteins were purified from the solution via Ni^2+^ immobilized metal ion affinity chromatography, using Cytiva HisTrap FF column (Cytvia; 17525501) and ÄKTA go protein purification system.

### SDS-PAGE and immunoblot

Protein samples were incubated at 80 °C for 10 min prior to loading on SDS-PAGE (10 or 15%) and run at 100–200 V until the dye reached the bottom of the gel. Proteins were transferred to either nitrocellulose or polyvinylidene difluoride (PVDF) membranes with a Tris–glycine transfer buffer (25 mM Tris, 192 mM glycine, 20% ethanol) for 90–120 min at 90 V or using the Bio-Rad Turbo blot system. Membranes with input samples were stained for total protein prior to immunoblotting (see ‘Total protein staining’). Membranes were blocked with 2% skimmed milk powder (Merck, cat. no. 70166) in PBS-T [phosphate-buffered saline (PBS) containing 0.1% Tween-20] before incubating with primary antibodies shaking for 1–2 h at room temperature, or overnight at 4 °C. The following antibodies were used: RFP (Chromotek, cat. no. 5F8), GFP (Santa Cruz Biotechnology, cat. no. sc-9996), myc (Santa Cruz, cat. no. sc-40), GST (Thermo Fisher Scientific, cat. no. MA4-004). Membranes were washed with PBS-T before incubating with respective secondary antibodies, mouse (Li-Cor, cat. no. 926-33210) or rat (Li-Cor, cat. no. 926-32219) in the dark at room temperature for 1 h. Proteins were detected with Li-Cor Odyssey CLx in the 700 and 800 nm channels.

### Total protein staining

After transfer, membranes were dried for at room temperature (1 h to overnight), rehydrated with ethanol (PVDF) or PBS (nitrocellulose) and stained with Revert™ 700 Total Protein Stain (Li-Cor, cat. no. 926-11021) or a homemade version [30% ethanol, 7% acetic acid, 0.001% Fast Green FCF (Thermo Fisher Scientific, cat. no. A16250)] for 10 min. Membranes were washed with Revert wash solution (30% ethanol, 7% acetic acid) to remove background and imaged using a Li-Cor odyssey CLx with the 700 nm channel. The stain was removed with Revert stain destain (30% ethanol, 100 mM sodium hydroxide). Membranes were rinsed with PBS before proceeding to blocking.

### Confocal imaging and image analysis

For Mp1 and Mp58 subcellular localization experiments, confocal microscopy was performed 3 d post-agroinfiltration. Live leaf tissue was mounted on a slide with the abaxial side facing upwards. Imaging was performed using a Zeiss LSM 710 with Plan Apochromat ×40/1.0 water dipping lens. The excitation wavelength for monomeric RFP was 561 nm and its emission was collected from 572 to 630 nm. GFP was imaged using 488 nm excitation, and its emission was collected from 500 to 530 nm. The pinhole was set to 1 AU for the shortest wavelength. Images were taken using line-by-line sequential scanning with 1024×1024 pixels with 2× line averaging.

For all other subcellular localization experiments, confocal microscopy was performed 2 d post-agroinfiltration. For this, the abaxial side of the live leaf tissue was mounted on a 1% agar pad moulded on a slide using a gene frame sealed with a coverslip [22 mm×22 mm #1.5 thickness (Thermo Scientific, cat. no. AB0577)]. Imaging was performed using a Nikon A1R confocal microscope with CF1 Plan apochromat VC ×60 water-immersion objective lens (NA 1.2, WD 0.27 mm, coverslip correction set to 0.17 mm). The laser excitation for BFP2, GFP, and RFP tags was at 405 nm (violet diode laser), 488 nm (argon laser, 10 mW), and 561 nm (sapphire laser, 20 mW), respectively. The emission ranges for BFP2, GFP, and RFP were 425–475 nm, 500–530 nm, and 570–620 nm, respectively. Autofluorescence from chlorophyll was captured at 488 nm and emissions collected between 663 and 738 nm. The pinhole was set to 1.2 AU for the shortest wavelength. Nyquist XY acquisition was performed for single optical slices, with zero offset and 5% laser power and a scanner zoom of 1.848 for all the channels. Galvano line-by-line sequential scanning was employed with a pixel dwell time of 2.4 frames per second, and at 1024×1024 pixels with 2× line averaging. All the images were captured using NIS-Elements software provided by the Nikon. GFP fluorescence was shown in yellow to ensure accessibility for colour blind readers. All data were imported to our OMERO server, using OMERO.Insight (Version: 5.7.2) for organization and annotation. Figures were generated in OMERO.Figure (Version: v4.4.3) (Allan *et al.*, 2012), with brightness and contrast adjusted where necessary. Region of interest-based intensity profile plots were generated in ImageJ–Fiji (Version: 1.54 g) using the plot profile plugin provided in the software.

### Protein structure and protein–protein interaction predictions

The protein sequences for Mp1 (XP_022169828.1) and Mp58 (XP_022169827.1) were submitted to SignalP 6.0, Phobius, and DeepTMHMM for signal peptide and transmembrane domain identification. By consensus a signal peptide was identified for Mp1 at amino acids (aa) 19–20 with probability 1.00 and cleavage probability 0.99 resulting in a 120 aa sequence and for Mp58 at aa 26–27 with probability 1.00 and cleavage probability 0.98 resulting in a 129 aa protein sequence. Signal peptide-encoding regions were removed and the resultant sequences submitted to IntFOLD-TS (Integrated Fold Recognition—Tertiary Structure) on the IntFOLD7 server (McGuffin *et al*., 2023, 2019), which uses an integrated trRosetta2 (Anishchenko *et al.*, 2021) and LocalColabFold 1.0.0 (Jumper *et al.*, 2021; Mirdita *et al.*, 2022) to predict protein secondary and tertiary structures. Global and local model quality estimates were carried out using ModFOLD9 (McGuffin *et al.*, 2021) and both single-chain and complex quality assessment were also conducted independently using DeepUMQA-X (Guo *et al.*, 2022), to assess local residue accuracy (local distance difference test, lDDT), complex interface accuracy [quaternary structure (QS) score], and folding accuracy [template modelling (TM) score].

Stereochemistry of deep-learning structural predictions was assessed using ProCheck incorporated in EMBL-EBI PDBsum (Laskowski *et al.*, 2018). This produced a whole-model Ramachandran plot and separate Ramachandran plots by residue for the Φ−ψ torsion angles of all residues, categorizing each combination of Φ−ψ due to each residues relative position to its neighbours as one of core, allowed, or disallowed. Side-chain torsion angle combinations (χ_1_−χ_2_), main-chain parameters including peptide bond planarity, Cα tetrahedral distortion, hydrogen-bond energy (Δ*E*_HB_) and overall G-factor were determined along with side-chain stereochemical parameters [σχ_1_ gauche(+), *trans*, and gauche(−) torsion angles, pooled σχ_1_, and finally σχ_2_  *trans* torsion angles]. To add to this analysis, main-chain bond lengths and angles, root mean square distances from planarity, and distorted geometry were determined.

Multimer predictions for, Mp1–Mp58, Mp1–AtVPS52, and Mp58–AtVPS52 and stoichiometric variations of combinations of these proteins were conducted using MultiFOLD (McGuffin *et al.*, 2023) and independently with AlphaFold3 (Abramson *et al.*, 2024) while quaternary structures were assessed using ModFOLDdock (Edmunds *et al.*, 2023; McGuffin *et al.*, 2023), AlphaFold3, DeepUMQA-X, and ProCheck.

### *Myzus persicae* performance assays on *N. benthamiana*

*Nicotiana benthamiana* plants were infiltrated with pAtSUC2::Mp1 and/or pAtSUC2::Mp58 under the phloem specific AtSUC2 promoter (pHW59) (Gottwald *et al.*, 2000). Two adult aphids were placed on the underside of leaves 1 d after agroinfiltration with clip cages. The following day adults were removed, and two 1st instar nymphs were left on each infiltration site. At 7 d post-infiltration, nymphs were transferred to new agroinfiltrated leaves and aphids were counted 7 d later (14 d post-initial agroinfiltration).

## Results

### *Myzus persicae* effectors Mp1 and Mp58 physically interact to form an effector complex

**Fig. 1.**
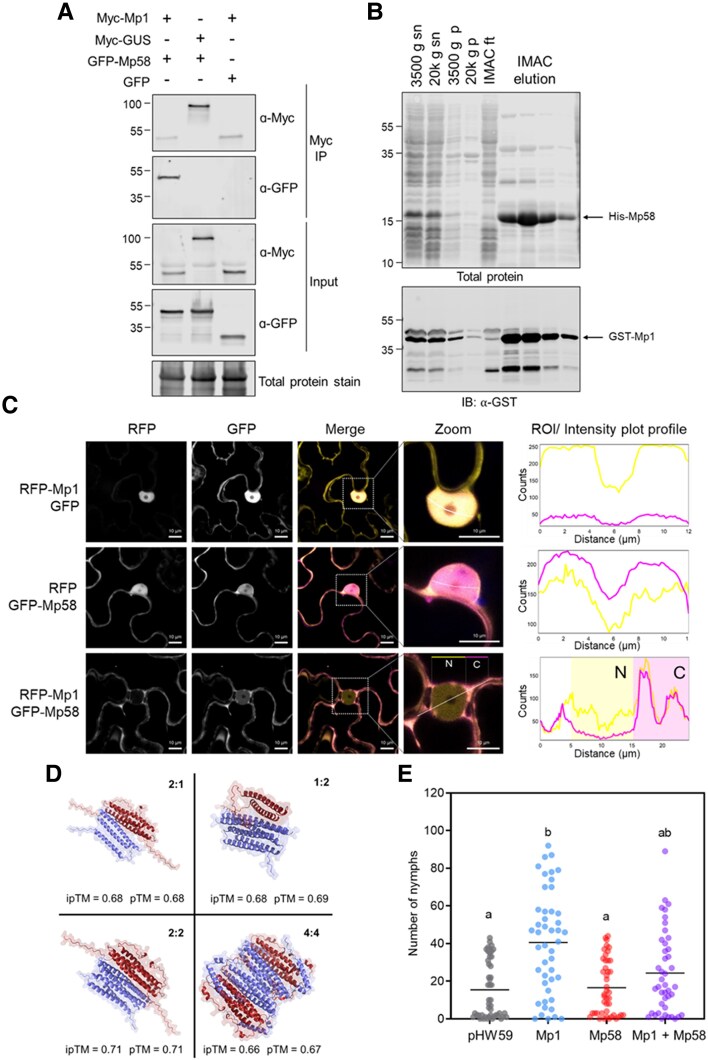
Mp1 and Mp58 interact *in vitro* and *in planta*. (A) Myc–Mp1 and GFP–Mp58 were transiently expressed via agroinfiltration with each other or GFP and myc–GUS as controls. Protein were immunoprecipitated with myc-trap. (B) Co-purification of His–Mp58 and GST–Mp1 from recombinant protein expressed in *E. coli*. Expression of His–Mp58 and GST–Mp1 was induced in *E. coli* individually and protein lysates were combined. Protein was purified via IMAC and blotted against GST. ft, flowthrough; p, pellet; sn, supernatant. Ladders represent kDa. (C) Co-localization of RFP–Mp1 (in magenta) and GFP–Mp58 (in yellow), and GFP or RFP, respectively, by confocal microscopy. Images were taken 3 d post-infiltration after agroinfiltration of *Nicotiana benthamiana*. Merged images represent overlay images of the GFP and RFP channels. White boxes in merge panels indicate zoomed section. The annotation at the bottom panel indicates the difference in the localization pattern of the proteins in nucleus (N) and cytosol (C). Scale bar 10 µm, magnification ×40. Presented images are single plane images. (D) AlphaFold3 structural predictions for Mp1–Mp58 hetero-oligomeric states. Predictions were conducted using AF3 manually by setting Mp1:Mp58 stoichiometric ratios within a range of all possible permutations from 1:1 to 4:4 and automatically using MultiFOLD2 (Supplementary Fig. S1). Shown are the four oligomeric combinations of Mp1 (red) and Mp58 (blue) with the highest ipTM scores as assessed by AlphaFold3, where Mp1:Mp58 in a 2:1 ratio resulted in an ipTM of 0.68 and pTM of 0.68; 1:2 ratio ipTM 0.68, pTM 0.69; 2:2 ratio ipTM 0.71, pTM 0.71; and 4:4 ratio ipTM 0.66, pTM 0.67. DeepUMQA-X analysis results in an interface lDDT of 0.626 for 2:1, 0.575 for 1:2, 0.652 for 2:2, and 0.656 for 4:4. (E) *Nicotiana benthamiana* leaves transiently expressing Mp1 and Mp58 under the phloem-specific promoter (AtSUC2) individually or together were challenged with *M. persicae* and fecundity was assessed over 14 d. Empty vector (pHW59) was used as a control. Empty vector pHW59 was included in individual effector samples (Mp1/Mp58 alone) to ensure the OD was consistent in all conditions. The graph shows all data points collected over three independent replicates (*n*=9–12). The horizontal line represents the mean. A Kruskal–Wallis test and Dunn’s multiple comparisons test was conducted for statistical analysis of differences between groups. Different letters represent *P*<0.01. dpi, days post-infiltration; GFP, green fluorescent protein; GUS, β-glucuronidase; IMAC, immobilized metal affinity chromatography; ipTM, interface predicted template modelling; lDDT, local distance difference test; pTM, predicted template modelling; RFP, red fluorescent protein; ROI, region of interest.

We previously showed that the effector genes encoding Mp1 and Me10-like (Mp58 in *M. persicae*) are paired and genetically linked across the genomes of five aphid species, and feature shared transcriptional control (Thorpe *et al.*, 2018). These observations led us to hypothesize that effectors Mp1 and Mp58 may function together during aphid infestation, potentially through protein–protein interaction. We first tested whether Mp1 and Mp58 can associate *in planta* by co-expressing myc–Mp1 with GFP–Mp58 in *N. benthamiana* followed by co-IP. We detected GFP–Mp58 in the myc–Mp1 immunoprecipitation, but not in the myc–GUS control, indicating that Mp1 and Mp58 associate (Fig. 1A). To determine whether the effector pair interact directly or whether the interaction may be mediated by interacting plant proteins, we produced recombinant GST–Mp1 and His–Mp58 proteins in *E. coli.* We individually expressed the proteins in *E. coli* and combined lysates before immobilized metal affinity chromatography (IMAC) of His–Mp58. Eluates were analysed by western blotting and showed co-purification of GST–Mp1, pointing to direct interaction between Mp1 and Mp58 (Fig. 1B).

We previously performed subcellular localization of GFP–Mp1 and GFP–Mp58, which showed a cytoplasmic/nuclear localization for both proteins (Rodriguez *et al*., 2017; Escudero-Martinez *et al*., 2020). While we initially generated a RFP–Mp58 construct for co-localization experiments, we were unable to consistently detect RFP–Mp58 by western blotting and confocal microscopy, while, in line with previous observations, GFP–Mp58 was easily detectable. Therefore, we performed co-localization experiments with the GFP–Mp58 and RFP–Mp1 combination. When individually expressed, RFP–Mp1 localizes predominantly to the nucleus, with a weaker signal observed in the cytoplasm (Fig. 1C). The weak RFP–Mp1 signal observed in the cytoplasm compared with GFP–Mp1 in previous reports (Rodriguez *et al.*, 2017; Escudero-Martinez *et al.*, 2020) is possibly due to lower expression of RFP–Mp1 compared with GFP–Mp1, though we were able to detect RFP–Mp1 by western blotting and detect an interaction between RFP–Mp1 and GFP–Mp58 via co-IP (Supplementary Fig. S1). GFP–Mp58, when expressed alone, localized to both the nucleus and cytoplasm (Fig. 1C) as previously described (Escudero-Martinez *et al.*, 2020). When RFP–Mp1 and GFP–Mp58 were co-expressed, GFP–Mp58 remained localized to the nucleus and the cytoplasm, whereas RFP–Mp1 was no longer present in the nucleus and only detected in the cytoplasm (Fig. 1C), suggesting that Mp1 relocalizes to the cytoplasm upon association with Mp58.

Computational modelling of the Mp1 and Mp58 structures with IntFOLD-TS, ModFOLD9, and DeepUMQA-X indicated that Mp1 and Mp58 both predominantly form α-helices (Supplementary Fig. S2). The computationally predicted structure of Mp1 [ModFOLD9: *P*=0.001, *E*=7.93e^−4^, global model quality score (GMQS) 0.571; DeepUMQA-X: TM score 0.734 and global lDDT 0.614] features a helix–turn–helix consisting of two interacting α-helices: a 34-residue helix at Thr23–Tyr56 (H1) and a 40-residue helix at Thr63–Thr102 (H2). There are 47 residue pairs that interact between the two helices (H1: 20, H2: 18, all located internal to their respective helices) with a distance-of-closest approach equal to 7.4 Å. The predicted Mp58 structure (ModFOLD9: *P*=0.01, *E*=7.26e^−3^, GMQS 0.4532; DeepUMQA-X: TM score 0.569 and global lDDT 0.566) consists of three α-helices and one 3_10_ helix: a 38-residue α-helix at Leu6–Phe43 (H1), which interacts with the 32-residue α-helix at Tyr49–Phe80 (H2), and the three-residue 3_10_ helix at Met98–Asn100 (H3), which interacts with the 18-residue α-helix at Asp102–Met119 (H4). There are a total of 48 internally sited interacting residue pairs between H1 and H2 (21 and 19 residues involved, respectively) with a distance-of-closest approach of 7.6 Å and a second helix–helix interaction between seven residue pairs of H2 and H4 (five residues from each helix) with a distance of 12.8 Å.

To understand how Mp1 and Mp58 interact to form an effector complex, we performed further modelling of the proteins using AlphaFold3, MultiFOLD, and DeepUMQA-X multimer quality analysis. Our models produced potential Mp1–Mp58 complex combinations with accuracy scores of 0.19–0.71 for interface predicted template modelling (ipTM) and 0.41–0.71 for predicted template modelling (pTM) (Supplementary Fig. S3). Analysis of the Mp1–Mp58 complex with a 1:2, 2:1, 2:2, and 4:4 ratio produced models with the highest confidence (Fig. 1D; Supplementary Fig. S4), indicating that Mp1 and Mp58 may form a larger oligomeric complex.

The Mp1–Mp58 modelled interaction with 1:2 stoichiometry identifies an interaction between three protein chains: A (Mp1), B, and C (Mp58) with a more involved interaction between the Mp58–Mp58 chains utilizing a total of 89 interface residues (Supplementary Fig. S5: chains B and C). This interaction has a total interface area of 2552 Å2 (chain B) and 2536 Å2 (chain C), in contrast to the Mp1–Mp58 interactions between chains A–B and A–C involving a total of 24 and 33 interface residues and an ∼26–38% chain-dependant interaction surface area respectively. In this model, both Mp58 H1 helices are aligned anti-parallel, and this is the case for H2–H2 and H3–H3 helices as well. The Mp1 H1 α-helix interacts with the Mp58(H1)–Mp58(H1) α-helix bundle at ∼45° forming five salt bridges and 14 hydrogen bonds (Supplementary Fig. S5A). A similar preference for Mp1–Mp1 interaction can be seen in the 2:1 stoichiometric model, where there are a total of 88 interface residues with an interaction surface area of 2199 Å^2^ and 2207 Å^2^ for each compared with the two Mp1–Mp58 interactions with 22 and 21 interface residues with an ∼29% of the interaction surface area. Each Mp1 α-helix aligns anti-parallel to the corresponding same α-helix in the interacting Mp1 chain (H1–H1, H2–H2) while Mp58 H1 interacts with the Mp1–Mp1, H1–H1 helical bundle at an angle of ∼45° with three salt bridges and eight hydrogen bonds (Supplementary Fig. S5B). The 2:2 heterodimer exhibits the same features where Mp1 and Mp58 interact more with other copies of the same protein, with each α-helix interacting with its counterpart in the opposing molecule in an anti-parallel fashion. The Mp1 H1–H1 helical pair interact with the Mp58 helical pair in the same manner as before at an ∼45° angle (Supplementary Fig. S5C). The 4:4 heterodimer interaction builds upon the 2:2 structure by the addition of a second 2:2 structure that is spatially antisymmetric (Supplementary Fig. S5D). Of note, Mp58–Mp58 H2–H2 interactions do not occur with the adjacent H2 but rather with the diagonally opposed H2 helix.

### Mp1 and Mp58 transient co-expression weakly enhances host susceptibility

Given the evidence Mp1 and Mp58 interact with one another, we investigated whether the combined expression of the effector pair alters host susceptibility to aphids. We previously showed that Mp1 ectopic expression in *N. benthamiana* enhanced susceptibility only when Mp1 was expressed under a phloem-specific AtSUC2 promoter (Rodriguez *et al*., 2017) while ectopic expression of Mp58 reduced aphid performance in transgenic Arabidopsis lines when expressed under a 35S and AtSUC2 promoter (Elzinga *et al*., 2014), and under a 35S promoter in *N. benthamiana* (Escudero-Martinez *et al.*, 2020). Therefore, we opted to use the phloem-specific AtSUC2 promoter to express Mp1 and Mp58 both individually and together in *N. benthamiana* and challenged infiltrated leaf areas with *M. persicae* to determine the combined impact of these effectors on host susceptibility. In line with previous work, Mp1 expression resulted in an increase in fecundity compared with the vector control (Fig. 1E). Expression of Mp58 did not result in a significant decrease in aphid fecundity compared with the vector control. When Mp1 and Mp58 were co-expressed, we observed a trend towards increased fecundity, but this increase was not statistically significantly different from the vector control (Fig. 1E).

### The Mp1-like and Mp58-like interaction is conserved in cereal aphid *Rhopalosiphum padi*

**Fig. 2.**
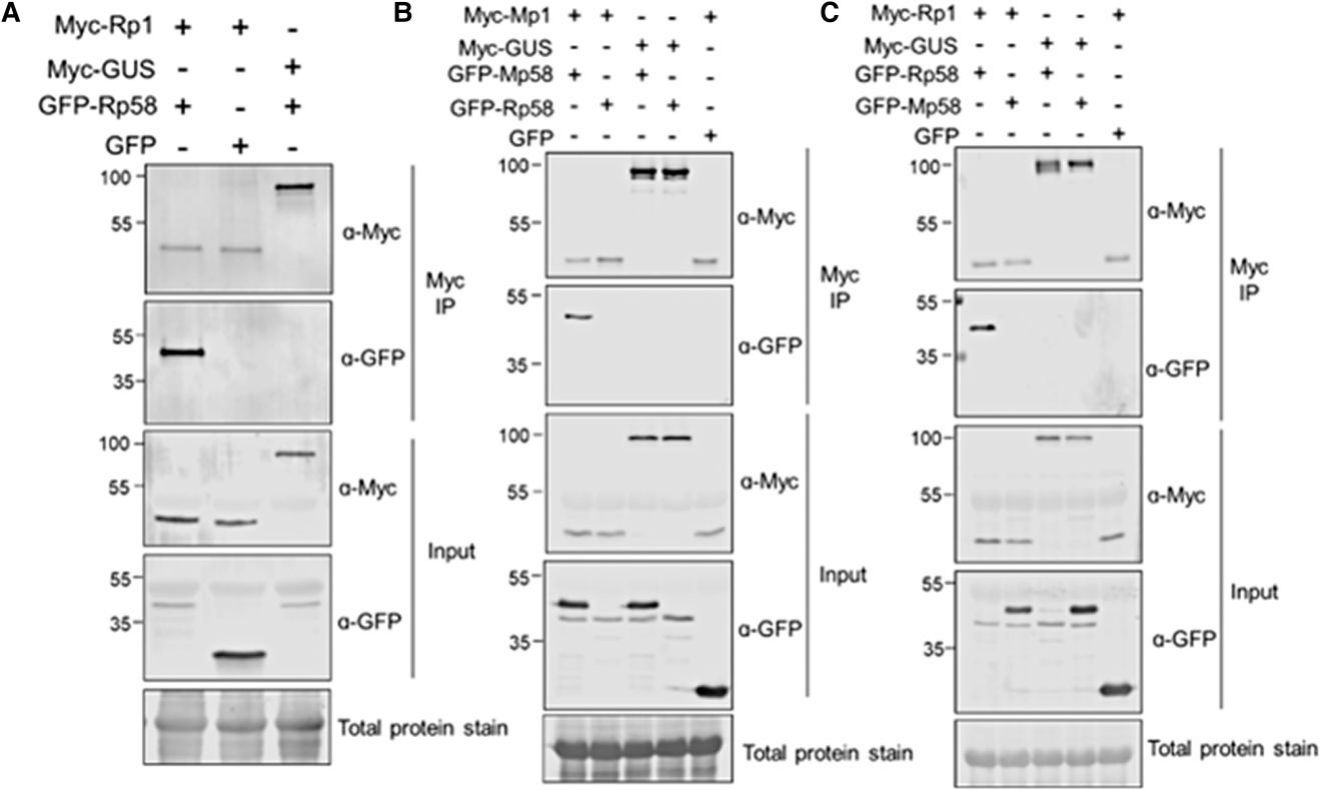
Mp1-like and Mp58-like interaction is conserved in the cereal aphid *Rhopalosiphum padi*, but interspecies interaction of the Mp1-like, Mp58-like effector pair is not detected. (A) Myc–Rp1 and GFP–Rp58 were transiently expressed via agroinfiltration with each other or GFP and myc–GUS as controls. Proteins were pulled down via myc-trap. (B, C) Myc–Mp1 (B) and Myc-Rp1 (C) were co-expressed in *N. benthamiana* with GFP–Mp58/GFP–Rp58 or GFP and myc–GUS as controls. Proteins were pulled down via myc-trap. Ladders represent kDa. GFP, green fluorescent protein; GUS, β-glucuronidase; IP, immunoprecipitation.

With the genetic linkage and shared transcriptional control of the Mp1–Mp58 pair being conserved across different aphid species (Thorpe *et al.*, 2018), we were interested to test whether Mp1-like and Mp58-like from other aphids can also interact. We co-expressed the putative Mp1/Mp58 orthologues from the aphid *Rhopalosiphum padi* (bird cherry-oat aphid) in *N. benthamiana* and performed co-IP to detect a possible interaction. We were able to detect GFP–Rp58 upon immunoprecipitation of myc–Rp1 but not in the myc–GUS negative control, indicating the interaction of the effector pair is conserved across aphid species (Fig. 2A).

With Rp1 and Mp1 showing 56% identity and Rp58 and Mp58 showing 65% identity (Escudero-Martinez *et al.*, 2020), we asked whether complex formation can take place with pair members across aphid species. We co-expressed myc–Mp1 or myc–Rp1 with GFP–Mp58 or GFP–Rp58 and performed a co-IP to test for interaction. We were only able to detect GFP–Mp58 but not GFP–Rp58 upon immunoprecipitation of myc–Mp1 (Fig. 2B). Due to lower expression levels of GFP–Rp58, we did not always detect GFP–Rp58 in the input blots when GFP–Mp58 and GFP–Rp58 were run side-by-side in SDS-PAGE and western blot experiments. Nevertheless, when we co-expressed myc–Rp1 with GFP–Rp58 or GFP–Mp58, we only detected GFP–Rp58 but not GFP–Mp58 upon immunoprecipitation of myc–Rp1 (Fig. 2C). Our observations point to species specificity in Mp1(-like) and Mp58(-like) complex formation, suggesting that the effectors have evolved together in each aphid species to mediate their activities.

### Mp1 and Mp58 are both able to associate with host protein VPS52

**Fig. 3.**
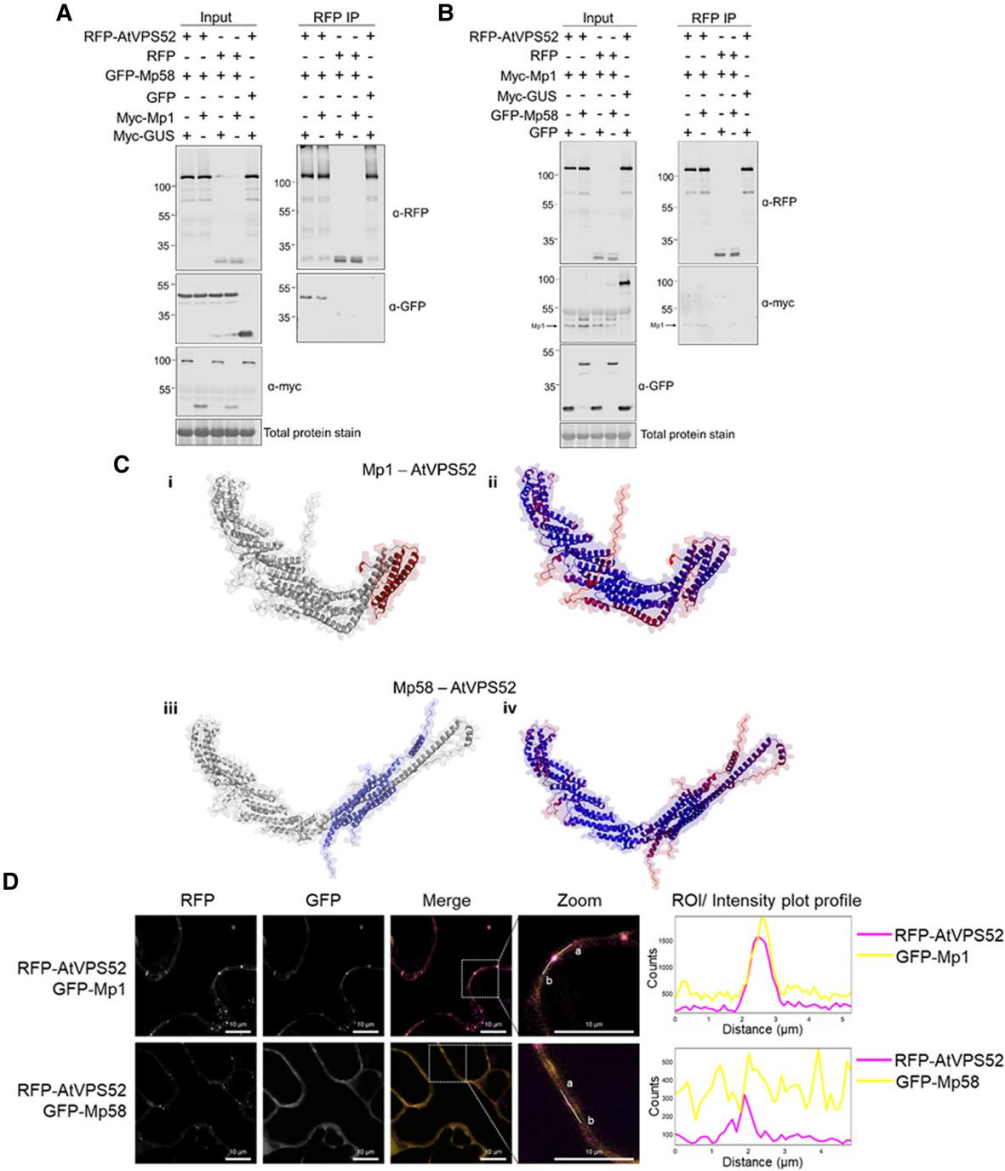
Mp1 and Mp58 both independently interact with AtVPS52, but only Mp1 localizes to AtVPS52-associated vesicles. Immunoprecipitation of RFP–AtVPS52 with (A) GFP–Mp58 in the presence of myc–Mp1 or myc–GUS (control) or (B) myc–Mp1 in the presence of GFP–Mp58 or GFP (control). Proteins were transiently expressed in *N. benthamiana* via agroinfiltration and pulled down with RFP-trap. Ladders represent kDa. (C) AlphaFold3 multimeric prediction of Mp1–AtVPS52 (i, ii) with 1:1 stoichiometry with AF3-derived ipTM 0.52, pTM 0.65, and DeepUMQA-X-derived TM score 0.816, QS score 0.701, global lDDT 0.665 and interface lDDT 0.632. (i) The Nʹ terminus of VPS52 (grey) interacting with Mp1 (red). (ii) The same interaction coloured by plDDT on a red–blue/low–high confidence scale. (iii, iv) AlphaFold3 multimeric prediction for Mp58–AtVPS52 with a 2:1 stoichiometry, with an AF3-derived ipTM 0.20, pTM 0.52 and DeepUMQA-X-derived TM score 0.748, QS score 0.371, global lDDT 0.677 and interface lDDT 0.642. (iii) A potential interaction between the Nʹ terminus of VPS52 (grey) and Mp58 (blue). (iv) The interaction coloured by plDDT. (D) Subcellular localization of RFP–AtVPS52 (in magenta) with GFP–Mp1 or GFP–Mp58 (in yellow). Proteins were transiently expressed *N. benthamiana* via agroinfiltration and localization was observed with confocal microscopy. Magnification of ×60 (water immersion lens), scale bar 10 µm (controls of individually expressed proteins in Supplementary Fig. S8). Presented images are single plane images. The merged panel transect corresponds to line intensity plot showing fluorescence distribution across the marked locus. GFP, green fluorescent protein; GUS, β-glucuronidase; IP, immunoprecipitation; ipTM, interface predicted template modelling; lDDT, local distance difference test; plDDT, predicted local distance difference test; pTM, predicted template modelling; QS, quaternary structure; RFP, red fluorescent protein; TM, template modelling.

We previously showed that Mp1 associates with VPS52 from Arabidopsis (AtVPS52) and *S. tuberosum* (StVPS52) in a species-specific manner and that this association is linked to Mp1 virulence activity (Rodriguez *et al.*, 2017). Since we found that Mp1 forms a complex with Mp58, and Mp1 and Mp58 share similar secondary structural features, we considered the possibility that Mp1 and Mp58 may share targets. We therefore tested whether Mp58, like Mp1, can associate with AtVPS52, and whether a possible association would be dependent on the presence of Mp1. GFP–Mp58 and RFP–AtVPS52 were co-expressed in *N. benthamiana* in the presence or absence of myc–Mp1 by agroinfiltration, followed by immunoprecipitation of RFP–AtVPS52. In both the presence and the absence of myc–Mp1, we were able to detect GFP–Mp58 upon immunoprecipitation of RFP–AtVPS52, indicating that Mp58 indeed associates with AtVPS52, and that this association is independent of Mp1 (Fig. 3A). The level of GFP–Mp58 that was detectable upon RFP–AtVPS52 pull-down was similar in the presence and absence of myc–Mp1, suggesting that the presence of Mp1 does not strengthen or weaken the association of Mp58 with AtVPS52. Likewise, we tested whether the interaction between Mp1 and AtVPS52 is altered in the presence of Mp58. We co-expressed myc–Mp1 and RFP–AtVPS52 in the presence or absence of GFP–Mp58. As expected, and in line with our previous work, myc–Mp1 was detected upon immunoprecipitation of RFP–VPS52 (Fig. 3B). The presence of GFP–Mp58 did not visibly affect the level of detectable myc–Mp1 in the RFP–VPS52 pull-down. Overall, these observations indicate that both Mp1 and Mp58 can interact independently with AtVPS52.

To determine potential Mp1–AtVPS52 and Mp58–AtVPS52 effector-target complex stoichiometries, we used MultiFOLD, which selected the stoichiometries autonomously as part of its deep-learning output, and produced multiple possible stoichiometries in AF3 from which the models with the highest ipTMs were selected. As VPS52 has been reported to interact with other GARP subunits in a 1:1:1:1 ratio in yeast (Conibear and Stevens, 2000; Siniossoglou and Pelham, 2002; Pérez-Victoria *et al.*, 2010), we used a single molecule of AtVPS52 while changing the number of Mp1 or Mp58 molecules. Predicted structural modelling of the Mp1–AtVPS52 interaction (AF3: ipTM 0.52 and pTM 0.65; DeepUMQA-X: TM score 0.816, QS score 0.701, global lDDT 0.665, interface lDDT 0.632) resulted in an interaction between the Nʹ-terminal portion of AtVPS52 and the helical bundle of Mp1 with a total of 76 interface residues, two salt bridges and six hydrogen bonds with a total interface area of 2283 Å^2^ of VPS52 and 2145 Å^2^ of Mp1. The interaction elicits a break in the α-helix of the Nʹ-terminal portion of VPS52 between residues aa 128–134, resulting in an H4 α-helix (E60–E128), β-turn at I129–I132 (IGSI), H5 3_10_ helix (S134–I136) and H6 α-helix (L137–I167) of which the portion beyond the Cʹ-terminus of the helix interacts with residues internal to H4 with a total of 34 interacting residues at a distance of 6.1 Å. The VPS52-H4 helix interacts with Mp1-H1 (22 residues, distance 1.9 Å) and Mp1-H2 (four residues, distance 10.7 Å) while VPS52-H6 predominantly interacts with Mp1-H2 (18 residues, distance 11.0 Å) then secondarily with Mp1-H1 (eight residues, 11.9 Å) (Fig. 3Ci, ii; Supplementary Figs S6A, S7A).

Modelling of the Mp58–AtVPS52 interaction (AF3: ipTM 0.20 and pTM 0.52; DeepUMQA-X: TM score 0.748, QS score 0.371, global lDDT 0.677, interface lDDT 0.642) produced the best ipTM and TM scores with a 2:1 stoichiometry. This interaction does not disrupt the N-terminal H4 α-helix, which remains a single helix spanning E60–I167. The Mp58–Mp58 complex retains the same structure as previously described and this interacts with VPS52 residues L9–D214, with the main interaction occurring at R154–D214 with a total of three salt bridges and eight hydrogen bonds, forming a AtVPS52 interface area of 3046 Å^2^ and 3080 Å^2^ with the Mp58–Mp58 complex (Fig. 3Ciii, iv; Supplementary Figs S6B, S7B).

### In contrast to Mp1, Mp58 does not relocalize to AtVPS52-associated vesicles

In plants, VPS52 has been found to localize to the Golgi and pre-vacuolar compartments (Lobstein *et al.*, 2004; Guermonprez *et al.*, 2008; Rodriguez *et al.*, 2017). In our previous work, we demonstrated that in the presence of AtVPS52 or StVPS52, Mp1 relocalizes to VPS52-associated vesicles (Rodriguez *et al.*, 2017). As we show that Mp58, similar to Mp1, interacts with AtVPS52 (Fig. 3A), we assessed whether Mp58 and AtVPS52 also co-localize. We co-expressed GFP–Mp58 or GFP–Mp1 (positive control) with RFP–AtVPS52 individually to determine subcellular location. As expected, RFP–AtVPS52 localized to the cytoplasm as well as to vesicle-like structures (Fig. 3D). GFP–Mp1 localized to the nucleus and cytoplasm, but when expressed alongside RFP–AtVPS52, relocalized to AtVPS52-associated vesicles (Fig. 3D; Supplementary Fig. S8). Interestingly, GFP–Mp58 localized to the nucleus and cytoplasm regardless of the presence of RFP–AtVPS52 (Fig. 3D; Supplementary Fig. S8), indicating that Mp58, unlike Mp1, does not relocalize to VPS52-associated vesicles but may associate with VPS52 in the cytoplasm instead. These observations suggest that although Mp1 and Mp58 both interact with AtVPS52, only the Mp1–AtVPS52 interaction occurs at VPS52-associated vesicles when we co-express with one effector at a time.

### Mp1 and Mp58 interact with different regions of AtVPS52

**Fig. 4.**
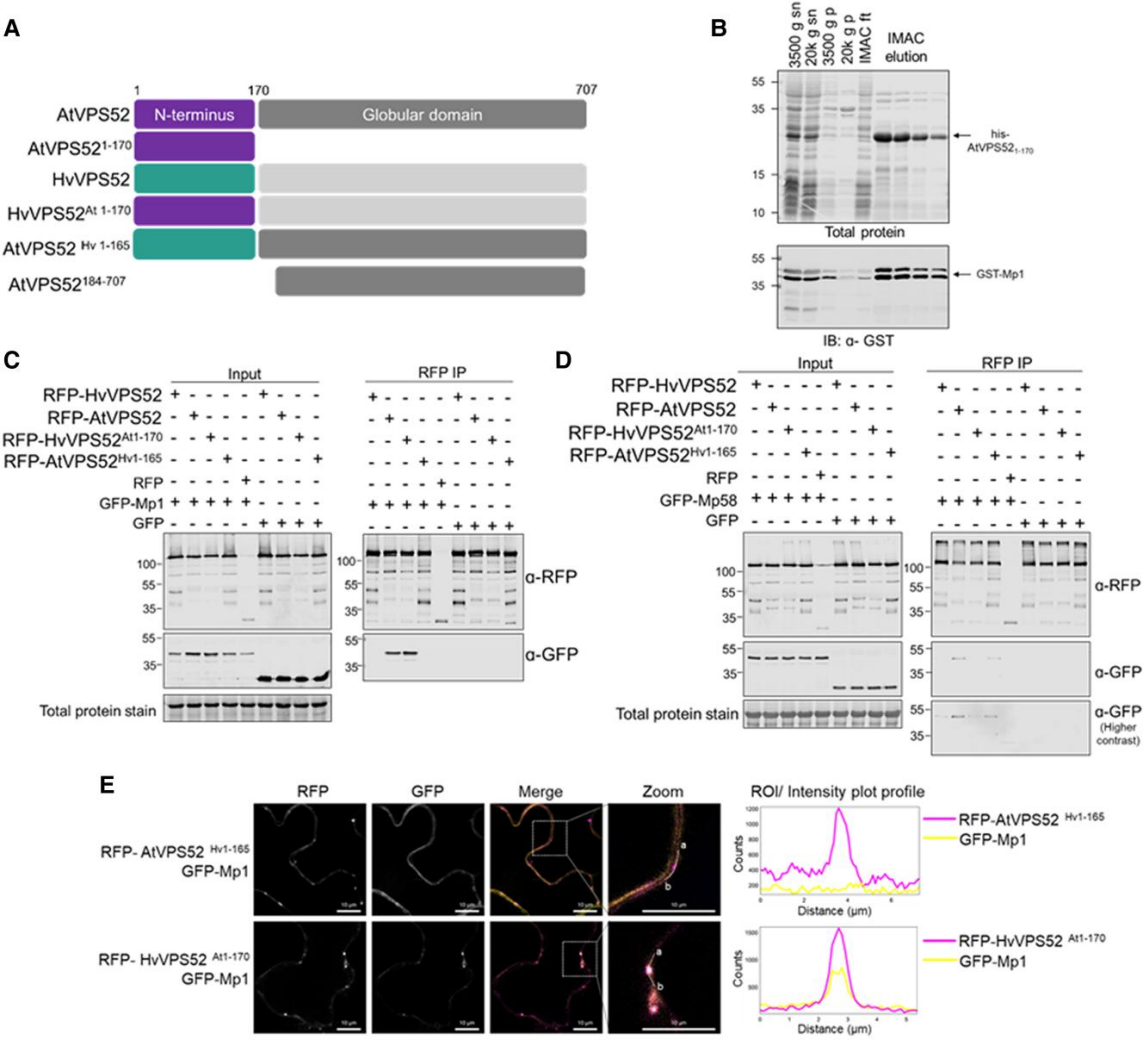
Mp1 and Mp58 interact with different regions of AtVPS52. (A) Schematic representation of full-length Arabidopsis (At) and barley (Hv) VPS52, and VPS52 variants. (B) Co-purification of His–AtVPS52^1–170^ with GST–Mp1 from recombinant protein expressed in *E. coli*. Expression of His- and GST-tagged proteins was induced in *E. coli* individually and protein lysates were combined. Protein was purified via IMAC and blotted against GST. ft, flowthrough; p, pellet; sn, supernatant. (C, D) GFP–Mp1 or GFP–Mp58 was co-expressed with RFP–HvVPS52, RFP–AtVPS52, or N-terminal chimeras in *N. benthamiana* via agroinfiltration. RFP-tagged proteins were pulled down with RFP-trap and blotted against GFP. RFP and GFP were used as negative controls. Ladders represent kDa. (E) GFP–Mp1 (in yellow) was co-expressed with RFP–AtVPS52^Hv1–165^ or RFP–HvVPS52^At1–170^ (in magenta) in *N. benthamiana* via agroinfiltration, and subcellular localization was observed with confocal microscopy. Magnification ×60 (water immersion lens), scale bar 10 µm (controls of individually expressed proteins, Supplementary Fig. S11). Presented images are single plane images. The merged panel transect corresponds to line intensity plot showing fluorescence distribution across the marked locus. GFP, green fluorescent protein; GST, glutathione *S*-transferase; IMAC, immobilized metal affinity chromatography; IP, immunoprecipitation; RFP, red fluorescent protein; ROI, region of interest.

Based on our predicted 3D protein structure models (Fig. 3C), we generated AtVPS52 constructs expressing the 170 N-terminal amino acids of the protein, which contains the predicted interaction site for Mp1 (AtVPS52^1–170^, Fig. 4A), as well as a set of VPS52 chimeras in which we swapped this N-terminal domain between AtVPS52 and HvVPS52. With the Mp58–AtVPS52 interaction site less defined by computational modelling, we focused our structure–function analyses on AtVPS52 in the context of the Mp1 interaction whilst including Mp58 in various interaction assays. We first assessed interaction of AtVPS52^1–170^ with effectors Mp1 and Mp58 *in vitro*. For this, we individually expressed His–AtVPS52^1–170^ and GST–Mp1 or His–Mp58 and GST–AtVPS52^1–170^ in *E. coli* and combined lysates before IMAC. Purification of His–AtVPS52^1–170^ resulted in the co-purification of GST–Mp1 (Fig. 4B). We were unable to consistently purify GST–AtVPS52^1–170^ with His–Mp58, suggesting that the Mp58–AtVPS52 features different structural requirements. Multiple attempts to detect the tagged AtVPS52^1–170^ protein domain when transiently expressed in *N. benthamiana* were unsuccessful, possibly due to low protein stability in plant cells. Therefore, to test for interaction of the N-terminal AtVPS52 domain with the effectors, we designed VPS52 chimeras where the N-terminal region of AtVPS52 was swapped with the N-terminal region of HvVPS52, which is unable to interact with Mp1 (Rodriguez *et al.*, 2017), and vice versa (Supplementary Fig. S9). We refer to these chimeras as HvVPS52^At1–170^ and AtVPS52^Hv1–165^, respectively (Fig. 4A). GFP–Mp1 or GFP–Mp58 was co-expressed with RFP-tagged full-length AtVPS52, HvVPS52, or the VPS52 chimeras in *N. benthamiana* followed by immunoprecipitation of RFP–VPS52 variants. As expected, GFP–Mp1 co-immunoprecipitated with RFP–AtVPS52 but not HvVPS52 (Fig. 4C). GFP–Mp1 also co-immunoprecipitated with RFP–HvVPS52^At1–170^, but not with RFP–AtVPS52^Hv1–165^ (Fig. 4C). Although GFP–Mp58 co-immunoprecipitated with RFP–AtVPS52 as expected (Fig. 4D), we observed either no or a weak interaction of GFP–Mp58 with RFP–HvVPS52 across repeated experiments (Fig. 4D; Supplementary Fig. S10), pointing to a potentially weak interaction between Mp58 and HvVPS52. Interestingly, GFP–Mp58 consistently co-immunoprecipitated with RFP–AtVPS52^Hv1–165^, while we observed either no interaction or a weak interaction with RFP–HvVPS52^At1–170^ across biological replicates (Fig. 4D; Supplementary Fig. S10). Our observations indicate that while the Mp1–VPS52 interaction is host-specific and N-terminus dependent, the Mp58–VPS52 interaction may be more undiscriminating.

To determine whether the VPS52 chimeras feature a similar subcellular localization to wild-type AtVPS52, we performed confocal microscopy and found that both RFP–HvVPS52^At1–170^ and RFP– AtVPS52^Hv1–165^ localized to vesicles (Supplementary Fig. S11), indicating that the chimeras are likely still functional proteins. Since only Mp1 and not Mp58 relocalized to wild-type AtVPS52-associated vesicles, we performed co-localization experiments of the VPS52 chimeras with Mp1 only. In line with previous observations (Rodriguez *et al.*, 2017), GFP–Mp1 localized to RFP–AtVPS52- but not RFP–HvVPS52-associated vesicles (Fig. 4E). When GFP–Mp1 was co-expressed with the RFP–VPS52 chimeras, we observed localization of GFP–Mp1 to RFP–HvVPS52^At1–170^- but not RFP– AtVPS52^Hv1–165^-associated vesicles (Fig. 4E), suggesting that Mp1 association with AtVPS52 indeed requires the AtVPS52 N-terminal region.

We also generated a construct that expresses AtVPS52 missing the 170 N-terminal amino acids (AtVPS52^184–707^) for co-expression experiments. However, RFP–AtVPS52^184–707^ did not localize to vesicles (Supplementary Fig. S12), suggesting that the N-terminal region of AtVPS52 is required for VPS52 association with vesicles and potentially its function in plant cellular trafficking. Overall, our data demonstrate that the N-terminal region of AtVPS52 is required for association with GFP–Mp1, highlighting that sequence variation within this region defines interaction specificity.

### An Mp1–Mp58–VPS52 multiprotein complex associates with vesicle-like structures

Although Mp1 and Mp58 can interact with AtVPS52 independently of each other, we show that these proteins also form an effector complex, raising the question as to whether the two effectors can associate with VPS52 at the same time to form a multi-effector–VPS52 complex.

**Fig. 5.**
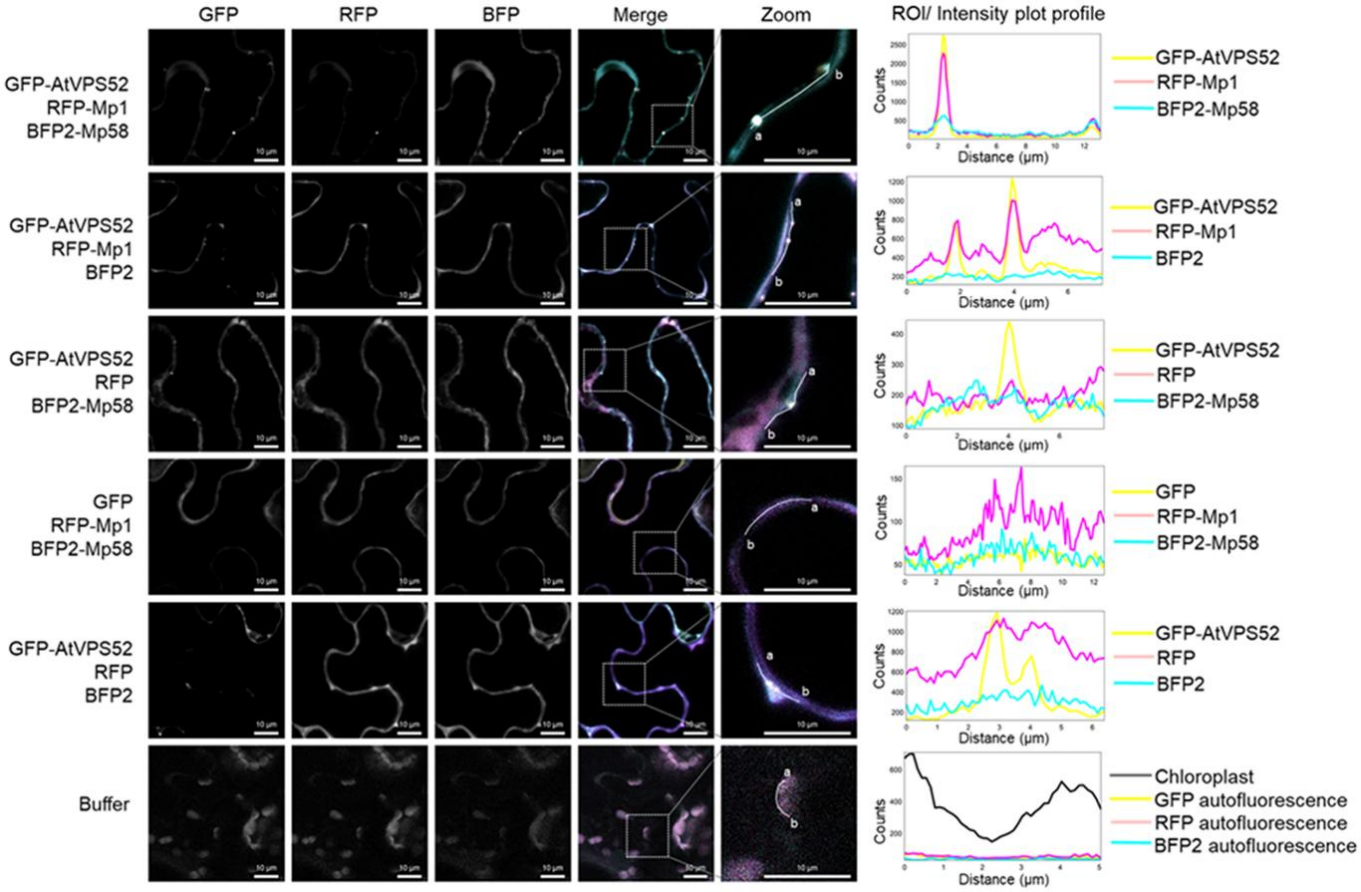
AtVPS52, Mp1, and Mp58 co-localize to vesicle-like structures. GFP–AtVPS52 (in yellow) was co-expressed with RFP–Mp1 (in magenta) and/or BFP2–Mp58 (in cyan) (GFP, RFP, and BFP2 used as controls). Proteins were expressed transiently in *Nicotiana benthamiana* and imaged at 2 dpi. Magnification ×60 (water immersion lens), scale bar 10 µm. Presented images are single plane images. The merged panel transect correspond to line intensity plot showing fluorescence distribution across the marked locus. BFP2, blue fluorescent protein; dpi, days post-infiltration; GFP, green fluorescent protein; RFP, red fluorescent protein.

We hypothesized that if indeed all three interact together, we would be able to detect the different proteins when co-expressed in plant cells in the same subcellular vesicle-like structure observed for AtVPS52. We transiently expressed GFP–AtVPS52 with BFP2–Mp58 and/or RFP–Mp1 (with GFP, BFP2, or RFP as controls) in *N. benthamiana* and observed subcellular localization using confocal microscopy. As previously shown, when AtVPS52 is expressed alone, we observed localization to the cytoplasm as well as to vesicle-like structures (Fig. 5). In line with our previous findings, we observed localization of RFP–Mp1 to GFP–AtVPS52-associated vesicle-like structures, while BFP2–Mp58 remained localized to the nucleus and cytoplasm when co-expressed with GFP–AtVPS52 (Fig. 5). Interestingly, when GFP–AtVPS52, BFP2–Mp58, and RFP–Mp1, were co-expressed we observed partial localization of Mp1, Mp58, and AtVPS52 to AtVPS52-associated vesicle-like structures (Fig. 5). This suggests that Mp1, Mp58, and AtVPS52 may indeed form a complex and that Mp58 only localizes to AtVPS52-associated vesicles in the presence of Mp1, pointing to potential effector cooperation.

## Discussion

Effectors play a crucial role in manipulating plant cell processes to facilitate pathogen infection and pest infestation. While pathogens and pests typically secrete a wide range of effectors into their host plants, studies on effector functions usually focus on individual proteins. Here, we demonstrate that effectors from an economically important aphid pest, *M. persicae*, form a complex to potentially work together, underscoring the importance of examining effectors within the context of the wider effector repertoire. We show that effectors Mp1 and Mp58, which are encoded by a physically linked and tightly co-regulated gene pair conserved across aphid genomes, interact to form an effector complex (Fig. 1). Together these effectors can bind to the previously identified Mp1 target AtVPS52 at vesicle-like structures (Fig. 5; Rodriguez *et al.*, 2017). Our findings point to effector complex formation in plant–insect interactions and highlight a yet-to-be-explored layer of complexity in the plant–insect molecular dialogue.

**Fig. 6.**
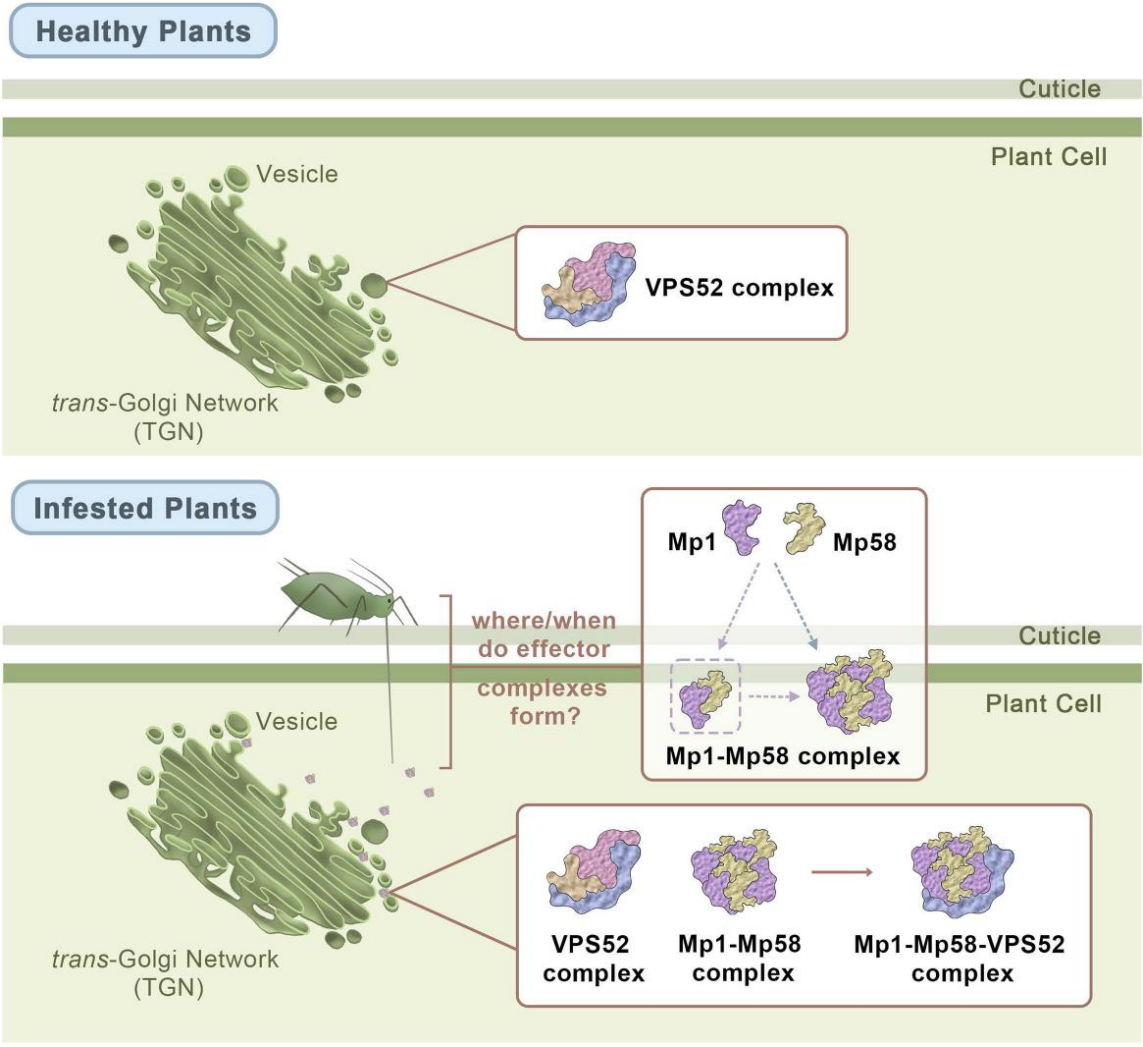
Working model of Mp1–Mp58–AtVPS52 complex. Upon infestation, *Myzus persicae* effectors Mp1 and Mp58 are secreted into host plant cells. Mp1 and Mp58 interact to form a complex, but the size and stoichiometry of the complex and whether the complex forms before, during, or after delivery remain to be investigated. Mp1 and Mp58 associate with VPS52-associated vesicles, which may disrupt or alter VPS52-associated complexes to alter vesicle trafficking mechanisms to benefit aphid feeding.

Effector complex formation has been reported in pathogens more broadly and was first described for effectors LubX and SidH in the human bacterial pathogen *Legionella pneumophila*. Effector LubX, a ubiquitin E3 ligase, targets the SidH effector for proteasome-mediated degradation to regulate its activity during infection stages (Kubori *et al.*, 2010). The term ‘metaeffector’ was proposed to describe effectors like LubX that regulate the activities of other effectors (Kubori *et al.*, 2010; Joseph and Shames, 2021). Extensive interaction analyses of ∼330–390 *L. pneumophila* effectors have since identified ∼20 interacting pairs pointing to extensive metaeffector activity (Urbanus *et al.*, 2016; Mount *et al.*, 2024). Similarly, some effectors from plant pathogenic microbes feature metaeffector activity, for example to suppress activation of effector-triggered immunity (Martel *et al.*, 2022). This can be achieved via protein–protein interactions with the same host protein, as is the case for several *Pseudomonas syringae* effectors (reviewed in Bundalovic-Torma *et al*., 2022). Further evidence for metaeffector activity in plant–pathogen interactions is provided by evidence that some effectors can form heteromeric complexes, which are suggested to be involved in regulation of effector translocation and/or activity (Ma *et al*., 2015; Li *et al*., 2016; Cao *et al*., 2018; Alcântara *et al*., 2019; Ludwig *et al*., 2021). Similar to the Mp1–Mp58 effector pair in *M. persicae*, interacting and/or cooperating effectors from *Fusarium oxysporum* f. sp. *lycopersici* (*Fol*) and f. sp. *conglutinans* are co-located; however, their expression seems to be divergent rather than co-regulated (Ma *et al.*, 2015; Ayukawa *et al.*, 2021; Yu *et al.*, 2024). Functional studies showed that *Fol* effector Six5 interacts with Avr2, and increases the plasmodesmatal size exclusion limit to facilitate the symplastic transport of Avr2, and potentially additional effectors, a function that is conserved amongst plant fungal pathogens (Cao *et al.*, 2018; Blekemolen *et al.*, 2022; Talbi *et al.*, 2023, 2024). While we demonstrate that Mp1 and Mp58 physically interact with one another as well as with the previously identified Mp1 target VPS52 (Figs 1, 3), the mechanistic role and composition of effector(–target) complex(es) remains to be addressed. Our observations raise new questions regarding when and where complex formation takes place, before, during, and/or after effector delivery (Fig. 6), and what the biological relevance of complex formation is. For example, Mp1–Mp58 complex formation may regulate individual effector activities towards certain host targets, regulate effector delivery, and/or allow diversification of potential host targets (i.e. complexes may interact with different host proteins from individual effectors). The individual Mp1 and Mp58 effector proteins are predicted to be relatively unstructured with two regions predicted to form α-helices, though these predictions have relatively low accuracy scores (Waksman *et al.*, 2024). Assuming a 1:1 interaction, computational modelling highlights an Mp1–Mp58 interaction interface between the predicted α-helices (Fig. 1). However, our computational analyses indicate that Mp1 and Mp58 likely form a larger oligomeric complex (Fig. 1).

Although the *Mp1–Mp58* gene pair is co-located and conserved across the genomes of at least five aphid species, with evidence for shared transcriptional control, our data show that the effector–effector interaction is species-specific (Fig. 2). The sequence variation of the Mp1 and Mp58 effector sequences across species (±55–65% sequence identity between the *M. persicae* and *R. padi* pair) suggests the effector pairs have evolved together within each aphid species and diversified across species due to functional adaptation in different host plants species. Either the host protein(s) targeted by the effector pair in different host species have a similar function but feature significant structural diversity or the effector pair may have evolved divergent functions in different hosts. Our previous work showed that Mp1 specifically targets VPS52 in host plant species; however, we were unable to detect interaction between Rp1 and HvVPS52 (Rodriguez *et al.*, 2017), which may suggest that Mp1-like and Mp58-like effector pairs have evolved different functions across plant species, though further experimental work will be required to explore this.

The co-localization of Mp1, Mp58, and AtVPS52 in vesicle-like structures upon co-expression (Fig. 5) is in line with a model that the two effectors and their target can associate on one complex (Fig. 6). Although both effectors also individually can associate with AtVPS52 (Figs 3, 5), the presence of Mp58 at AtVPS52-associated vesicle-like structures only occurs in the presence of Mp1. This raises the question whether Mp1 may be acting as a chaperone to Mp58 and/or facilitating its activity. In the case of the *Fol* Avr2–Six5 pair, the movement of Avr2 between cells is significantly increased in the presence of Six5, showing that an important role of Six5 is to facilitate Avr2 movement and activity (Blekemolen *et al.*, 2022). Whether, and if so, how Mp1 or Mp58 similarly regulates movement and/or activity of the other effector pair member remains to be investigated.

Additionally, Mp1 and Mp58 interact with different regions of AtVPS52, indicating that these effectors may simultaneously target the same host protein by binding to distinct domains (Fig. 4). We did not find evidence for competitive binding of Mp1 and Mp58 to VPS52 in co-IP assays, which is in line with these effectors binding to different AtVPS52 regions. Although we previously showed the VPS52 overexpression increases host immunity to aphids (Rodriguez *et al*., 2017), the mechanisms by which Mp1 and Mp58 target VPS52 and the downstream consequences of this remain to be revealed. Within the GARP complex VPS52 associates with the subunits VPS51, VPS53 and VPS54, and in *Saccharomyces cerevisiae* these interactions involve the N-terminal α-helices of these proteins, as determined by electron microscopy (Chou *et al.*, 2016). However, most of these subunits, including VPS52, can also associate with additional cellular trafficking complexes, such as the EARP (endosome-associated recycling protein) complex, which is involved endocytic recycling as shown in human cells and yeast (Schindler *et al.*, 2015). We hypothesize that Mp1 and/or Mp58 may disrupt or modify one of these cellular trafficking complexes through association with VPS52 to benefit aphid infestation (Fig. 6). In line with this, we previously found that AtVPS52 was degraded post-transcriptionally upon aphid infestation; however, this observation could not be attributed to Mp1 based on transient ectopic expression-based assays (Rodriguez *et al.*, 2017). We did not observe any reduction in VPS52 levels upon co-expression with Mp58 or the Mp1–Mp58 combination. It is possible that additional aphid effectors are involved in targeting VPS52, potentially independent of the Mp1–Mp58 effector pair. Additional approaches to identify novel targets of the Mp1–Mp58 complex and investigate how the Mp1–Mp58 complex may affect cellular trafficking via VPS52, for example via endosome localization and/or protein secretion, would provide insight into the downstream consequences of the Mp1–Mp58 complex targeting VPS52 and how this involves effector cooperation.

The lack of a strong virulence phenotype upon combined expression of Mp1 and Mp58 (Fig. 1) may point to a role of effector complex formation in perhaps effector activity regulation rather than cooperation. However, the functional assays available to test whether *M. persicae* effectors contribute to promoting susceptibility have significant limitations as they rely on ectopic expression of aphid proteins in plants. It is well established that ectopic expression of effectors in plants may result in excessive targeting or mis-targeting of host proteins. This can lead to different or even opposite phenotypes to those expected based on actual virulence activity, which is in part due to a lack of knowledge on endogenous effector amounts and spatiotemporal effects of effector expression during aphid infestation. Moreover, our combined effector expression assay requires co-transformation of the same plant cells using *Agrobacterium* carrying different constructs, which may dilute any observable phenotypes. In previous studies (Pitino and Hogenhout, 2013; Rodriguez *et al.*, 2017), expression of Mp1 alone increased host susceptibility to aphids, whereas expression of Mp58 reduced host susceptibility. Differences in experimental conditions, such as the expression of Mp58 under the AtSUC2 promoter in *N. benthamiana*, which has not been previously tested, could explain the lack of an Mp58 phenotype in our assays. With no genetic modification system currently established for *M. persicae* and RNAi resulting in low or inconsistent levels of reduced expression, options to reliably screen for aphid effector virulence activities are limited, with further tool development needed to help advance the field. Characterization of the host targets of effectors like Mp1 and Mp58 will help us to explore their extended phenotype in promoting host susceptibility. For example, in previous work we showed VPS52 contributes plant immunity to aphids in transient expression assays and that this target protein is degraded during aphid infestation. Unfortunately, homozygous knock-out mutants for *VPS52* as well as other GARP complex subunits cannot be obtained due to lethality/gametophyte defects, limiting further genetic approaches in Arabidopsis. However, gene editing strategies to target specific *AtVPS52* regions, for example required for Mp1 and/or Mp58 interaction, may help us further understand its role in plant–aphid interactions.

In conclusion, this work provides evidence that effectors from phloem-feeding insects can associate with one another to form effector complexes as well as with (shared) host proteins, potentially to promote infestation. Our data point to effector complex formation in plant–insect interactions and raise new research questions about the role of effector complex formation in regulating and/or diversifying effector activities, and whether effector complex formation is a common feature within herbivorous insect effector repertoires. Overall, the observation that proteins encoded by a conserved and genetically linked effector gene pair can form an effector complex highlights a yet-to-be-explored layer of complexity to consider in the molecular dialogue between plants and herbivorous insects.

## Supplementary Material

erag070_Supplementary_Data

## Data Availability

Additional replicates and uncropped western blots are provided in Supplementary Fig. S13. The primary data supporting this study were not made publicly available at the time of publication. The data that support the findings of this study are available from the corresponding author upon request.

## Supplementary data

The following supplementary data are available at *JXB* online.

Fig. S1. Co-immunoprecipitation of RFP–Mp1 and GFP–Mp58.

Fig. S2. Computational modelling of Mp1 and Mp58.

Fig. S3. Computational modelling of different Mp1–Mp58 complex conformations.

Fig. S4. AlphaFold3 and DeepUMQA-X quality analysis of the top four Mp1–Mp58 multimer predictions.

Fig. S5. PDBSum analysis of Mp1–Mp58 multimer predictions for the top four stoichiometries with the highest confidence.

Fig. S6. AlphaFold3 multimeric prediction of Mp1–AtVPS52 and Mp58–AtVPS52.

Fig. S7. PDBsum analysis of Mp1 or Mp58 and Vps52 multimer predictions.

Fig. S8. Subcellular localization of RFP–AtVPS52, GFP–Mp1, and GFP–Mp58 expressed with GFP or RFP (controls for Fig. 3D).

Fig. S9. Amino acid alignment of Arabidopsis VPS52 (AtVPS52) and barley VPS52 (HvVPS52).

Fig. S10. Individual replicates of co-immunoprecipitation of RFP–VPS52 variants and GFP–Mp58.

Fig. S11. Subcellular localization of RFP–VPS52 variants and GFP–Mp1.

Fig. S12. Subcellular localization of RFP–AtVPS52 N-terminal deletion mutant AtVPS52^184–707^.

Fig. S13. Uncropped western blots for Figs 1–4 including additional replicates.
