# Supplementary material for: Aphid effector pair Mp1–Mp58 forms an effector complex that targets a host trafficking protein
Source: J Exp Bot. 2026 Feb 21;77(12):3880–95. doi: 10.1093/jxb/erag070 (PMC13293125; doi:10.1093/jxb/erag070)
Supplement: erag070_Supplementary_Data [file erag070_supplementary_data.pdf]

**Supplementary data**

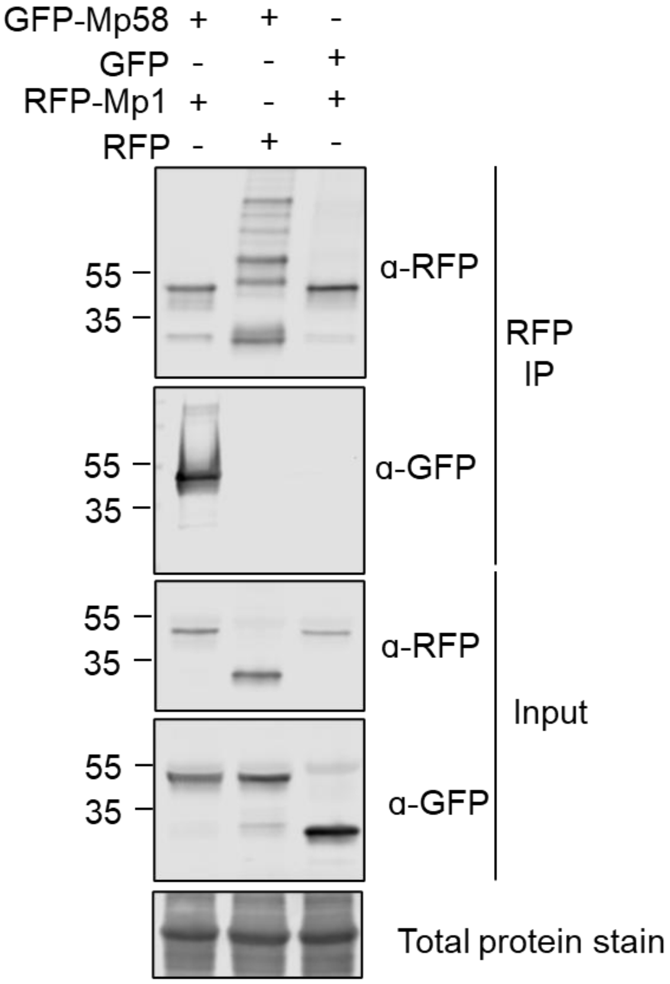


**Supplementary Figure S1:** co-immunoprecipitation of RFP-Mp1 and GFP-Mp58. RFP-Mp1 and GFP-Mp58 were transiently expressed via agroinfiltration with each other or GFP and RFP as controls. Proteins were immunoprecipitated with RFP-trap and blotted against RFP and GFP antibodies.

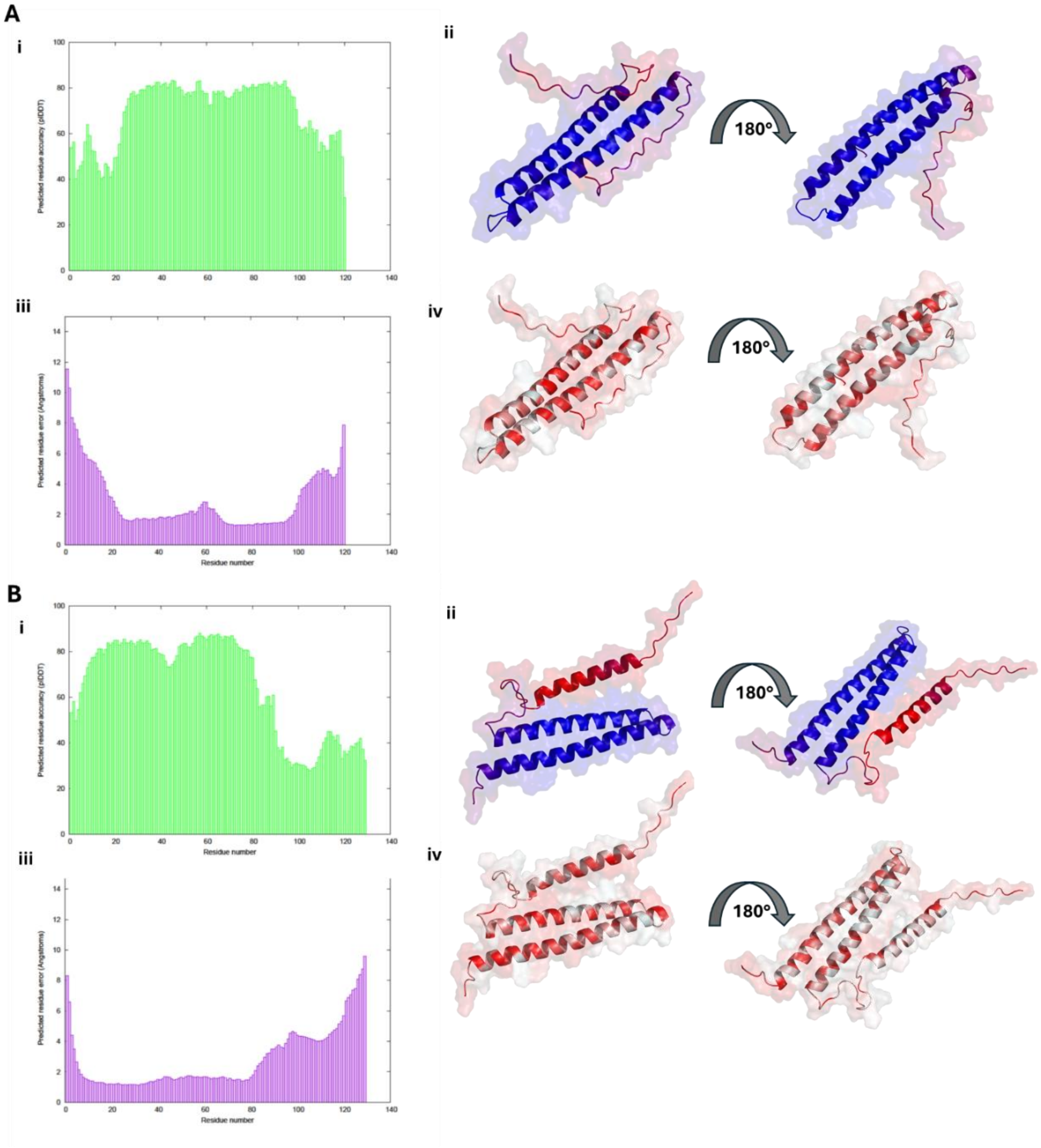


**Supplementary Figure S2: (A)** IntFOLD7 monomeric structural prediction of Mp1 produced a model with E = 7.93e$^{-4}$, p = 0.001, GMQS of 0.571 and DeepUMQA-X derived global lDDT of 69.07. Figure **(i)** displays the predicted residue accuracy (plDDT) and **(ii)** shows this mapped onto Mp1 using a red-blue/low-high scale. **(iii)** displays the predicted residue error in Å. **(iv)** shows the by-residue hydrophobicity of the model, indicating intra-chain interaction and a solvent-exposed series of residues along a second side of both α-helices. **(B)** IntFOLD7 monomeric structural prediction of Mp58 produced a model with E = 1.3e$^{-3}$, p = 0.01, GMQS of 0.545 and DeepUMQA-X derived global lDDT of 58.86. Figure **(i)** displays the predicted residue accuracy (plDDT) and **(ii)** shows this mapped onto Mp58 using a red-blue/low-high scale. The overall plDDT is reduced due to uncertainty over the location of the C' α-helix. **(iii)** displays the predicted residue error in Å. **(iv)** shows the by-residue hydrophobicity of the model, indicating intra-chain interaction between the first and second α-helices and a solvent-exposed series of residues along a second side each. The C' α-helix has a single hydrophobic side.

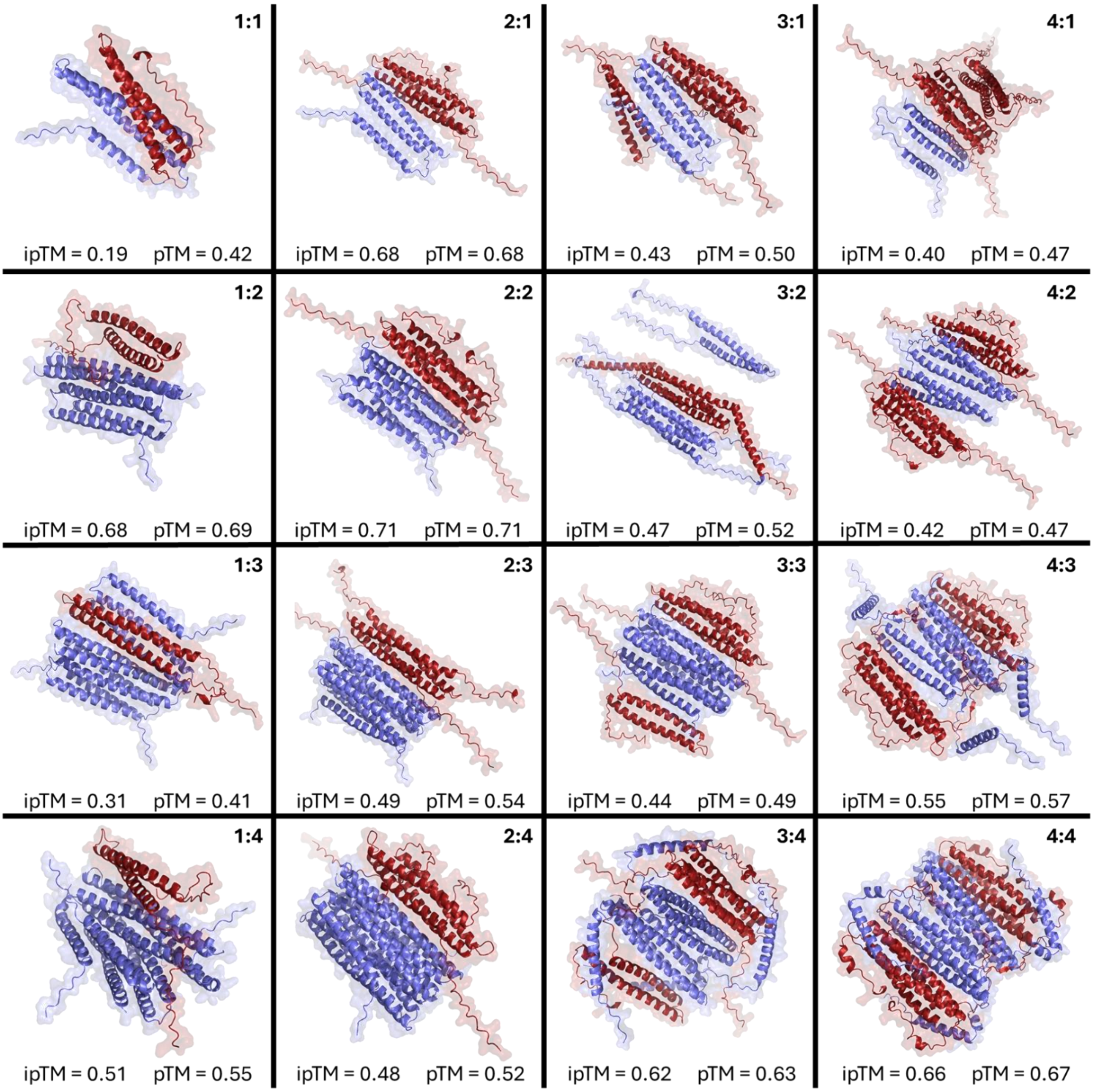


**Supplementary Figure S3:** Presented are 16 possible stoichiometric permutations of the Mp1+Mp58 interaction predicted using AlphaFold3. Predicted template modelling scores (pTM) which quantify the overall accuracy of the predicted protein complex structure ranged between 0.41 and 0.71, where values > 0.5 are regarded as similar to the true structure. The interface predicted template modelling Score (ipTM) evaluates and scores the accuracy of the predicted interaction interfaces in a protein-protein complex. This ranged between 0.19 and 0.71, where > 0.8 denotes high confidence in the predicted interface, 0.6 – 0.8 moderate confidence and < 0.6 low confidence. Due to the inclusion of disordered and non-interacting domains in the input sequences used in whole-model generation, ipTM scores are consequently lower than had only the interacting domains been modelled.

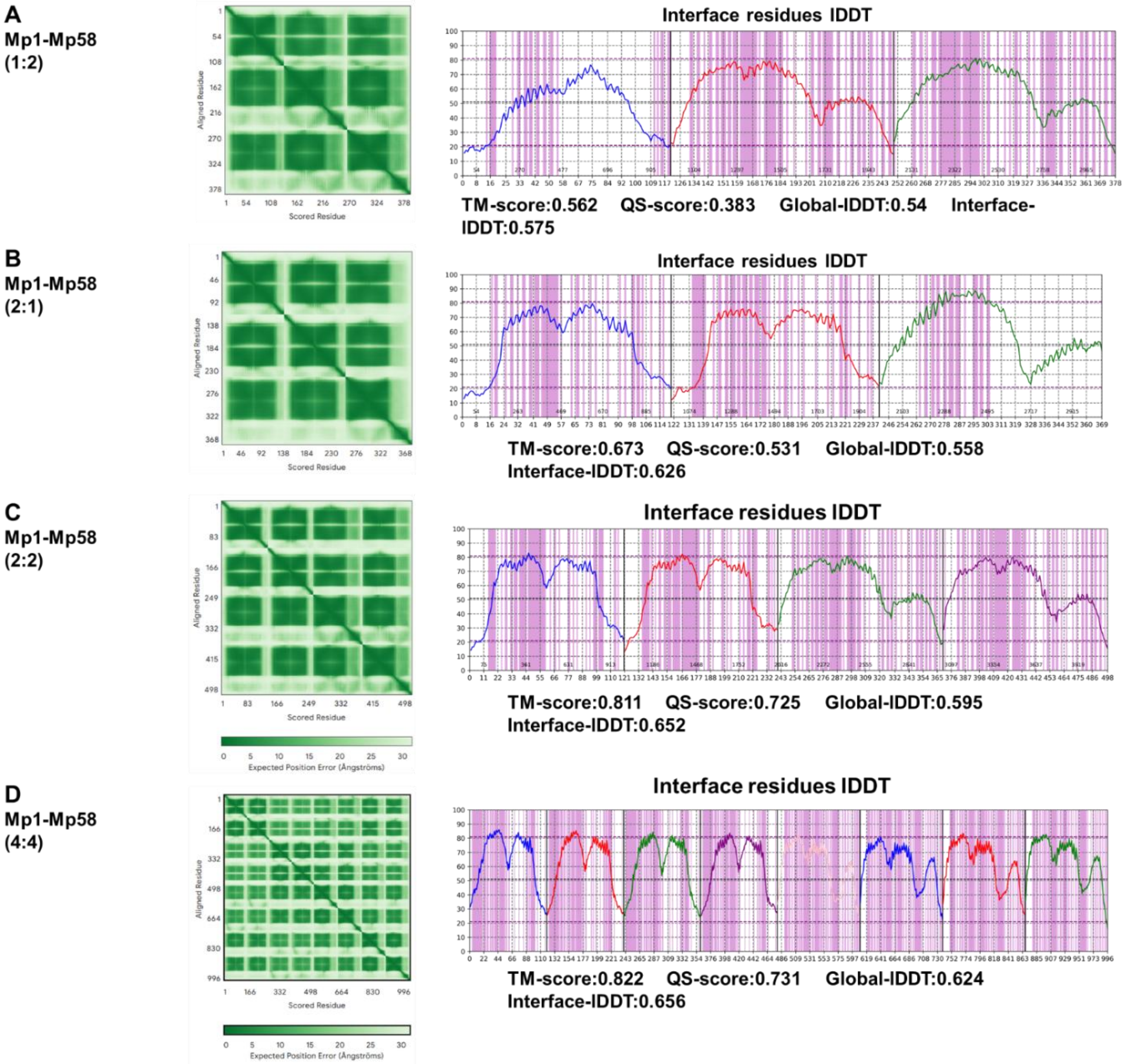


**Supplementary Figure S4:** AlphaFold 3 expected position error and DeepUMQA-X model quality analysis for highest ipTM and pTM Mp1-Mp58 interaction predictions with **(A)** 1:2, **(B)** 2:1, **(C)** 2:2, and **(D)** 4:4 stoichiometries.

**A**

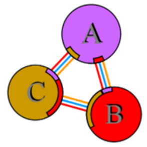


### Mp1:Mp58 1:2

| Chains | Interface Residues | Interface Area (Å²) | Salt bridges | Disulphide bonds | Hydrogen bonds |
|---|---|---|---|---|---|
| A B | 10 : 14 | 661 : 640 | 1 | - | 3 |
| A C | 17 : 16 | 972 : 944 | 4 | - | 11 |
| B C | 45 : 44 | 2552 : 2536 | 2 | - | 7 |

**B**

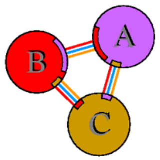


### Mp1:Mp58 2:1

| Chains | Interface Residues | Interface Area (Å²) | Salt bridges | Disulphide bonds | Hydrogen bonds |
|---|---|---|---|---|---|
| A B | 44 : 44 | 2199 : 2207 | 6 | - | 19 |
| A C | 11 : 11 | 612 : 606 | 2 | - | 7 |
| B C | 9 : 12 | 672 : 606 | 1 | - | 1 |

**C**

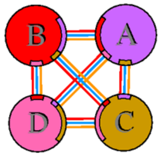


### Mp1:Mp58 2:2

| Chains | Interface Residues | Interface Area (Å²) | Salt bridges | Disulphide bonds | Hydrogen bonds |
|---|---|---|---|---|---|
| A B | 45 : 46 | 2361 : 2366 | 6 | - | 24 |
| A C | 11 : 16 | 779 : 745 | 1 | - | 2 |
| B D | 11 : 15 | 772 : 743 | 1 | - | 2 |
| A D | 12 : 11 | 631 : 616 | 2 | - | 8 |
| B C | 12 : 11 | 633 : 618 | 2 | - | 8 |
| C D | 43 : 44 | 2245 : 2253 | 4 | - | 3 |

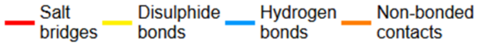


**D**

### Mp1:Mp58 4:4

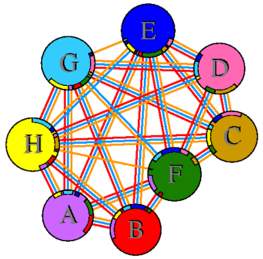


| Chains | Interface Residues | Interface Area (Å²) | Salt bridges | Disulphide bonds | Hydrogen bonds |
|---|---|---|---|---|---|
| A B | 36 : 36 | 2020 : 2020 | 6 | - | 14 |
| C D | 36 : 37 | 2026 : 2025 | 6 | - | 15 |
| A E | 5 : 8 | 338 : 296 | 1 | - | 2 |
| B F | 4 : 7 | 302 : 276 | 1 | - | 2 |
| C G | 5 : 8 | 348 : 305 | 1 | - | 3 |
| D H | 5 : 7 | 300 : 276 | 1 | - | 3 |
| A F | 18 : 20 | 1215 : 1243 | 1 | - | 8 |
| B E | 19 : 22 | 1265 : 1284 | 1 | - | 8 |
| C H | 18 : 20 | 1228 : 1237 | 1 | - | 8 |
| D G | 19 : 21 | 1287 : 1310 | 2 | - | 9 |
| A G | 8 : 10 | 707 : 676 | 1 | - | 2 |
| B H | 9 : 11 | 740 : 712 | 1 | - | 2 |
| C E | 8 : 9 | 708 : 686 | 1 | - | 2 |
| D F | 9 : 11 | 733 : 701 | 1 | - | 2 |
| A H | 12 : 10 | 613 : 608 | 3 | - | 5 |
| B G | 12 : 10 | 609 : 607 | 3 | - | 5 |
| C F | 12 : 10 | 612 : 608 | 3 | - | 5 |
| D E | 12 : 10 | 608 : 608 | 3 | - | 5 |
| E F | 32 : 31 | 1618 : 1619 | 4 | - | 4 |
| G H | 33 : 31 | 1626 : 1622 | 4 | - | 4 |
| E G | 2 : 2 | 191 : 196 | - | - | - |
| E H | 13 : 13 | 842 : 811 | - | - | 1 |
| F G | 13 : 13 | 815 : 852 | - | - | 3 |
| F H | 1 : 1 | 36 : 36 | - | - | - |

**Supplementary Figure S5:** PDBsum analysis of Mp1-Mp58 multimer predictions for varying stoichiometries. Part **(A)** provides the data for 1:2 stoichiometry with chains A representing Mp1 with B and C as Mp58. **(B)** shows 2:1 with Mp1 as chains A and B while C is Mp58. **(C)** shows a 2:2 ratio with A+B as Mp1 and C+D as Mp58. Figure **(D)** shows a 4:4 ratio with A+B+C+D as Mp1 and E+F+G+H as Mp58.

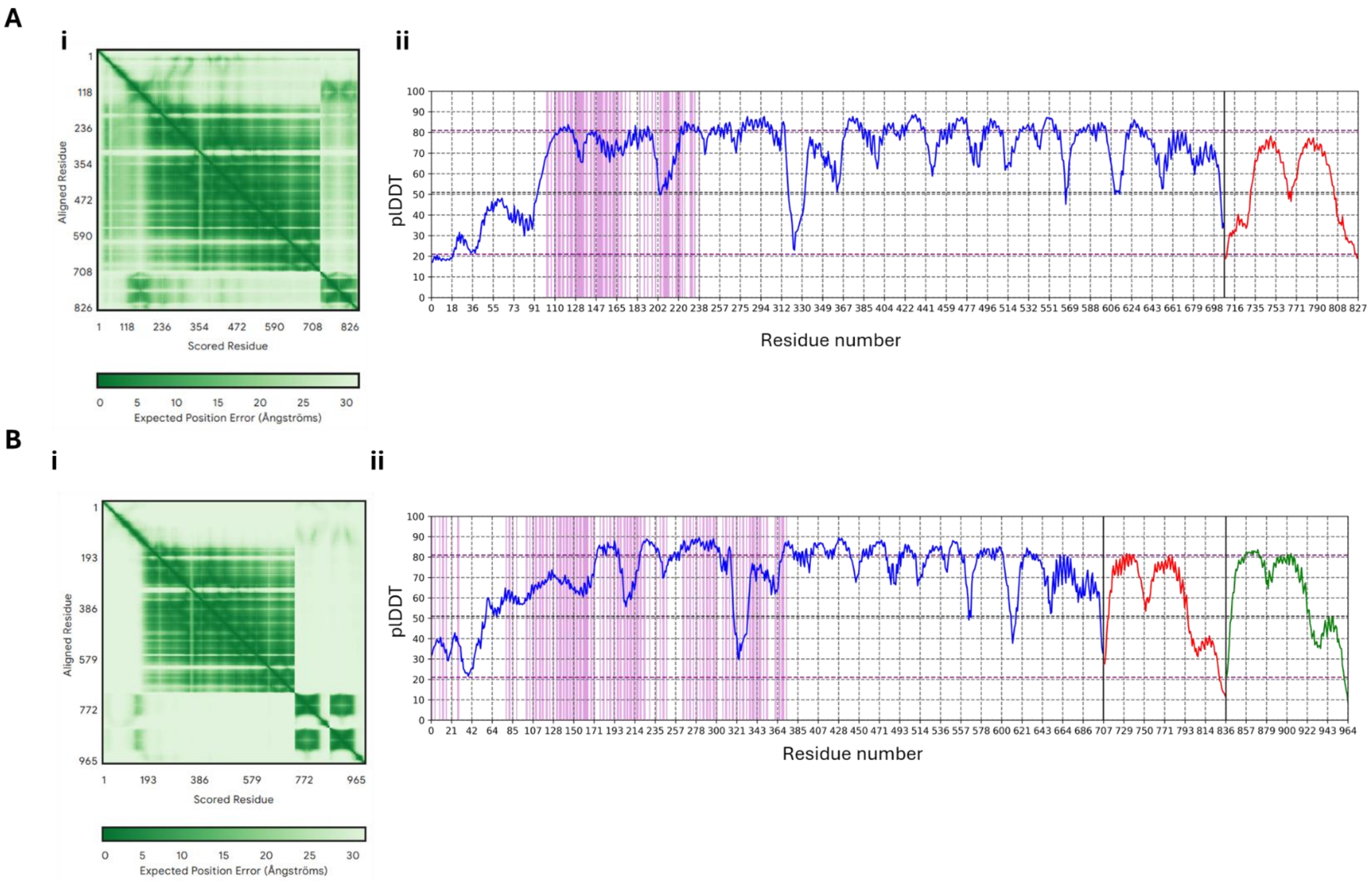


**Supplementary Figure S6: (A)** AlphaFold3 multimeric prediction of Mp1-AtVPS52 with 1:1 stoichiometry produced a model where ipTM = 0.52 and pTM = 0.65. **(i)** displays the position error in Å **(ii)** DeepUMQA-X results by residue, with a whole-model TM-score: 0.816, QS-score: 0.701, Global-lDDT:0.665 Interface-lDDT: 0.632 with highlighted VPS52 contact residues in magenta **(B)** AlphaFold3 multimeric prediction of Mp58-AtVPS52 with 2:1 stoichiometry produced a model where ipTM = 0.20 and pTM = 0.52. **(i)** displays the position error in Å **(ii)** DeepUMQA-X results by residue, with a whole-model TM-score: 0.748, QS-score: 0.0.371, Global-lDDT:0.677 Interface-lDDT: 0.642 with highlighted VPS52 contact residues in magenta

**A Mp1:Vps52 (1:1)**

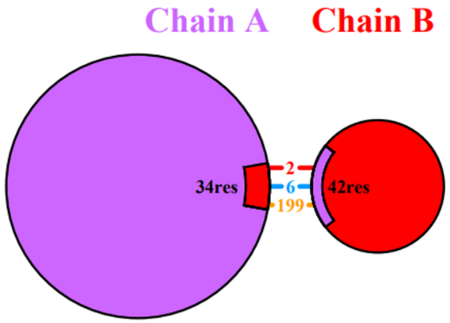


| Chain | Interface Residues | Interface area (Å2) | Salt bridge | Disulphide Bonds | Hydrogen Bonds |
|---|---|---|---|---|---|
| A | 34 | 2283 | 2 | - | 6 |
| B | 42 | 2145 | | | |

**B Mp58:Vps52 (2:1)**

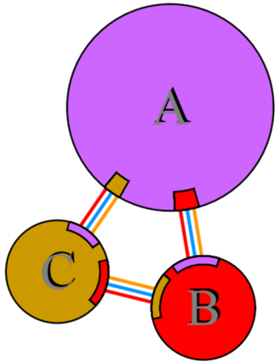


| Chain | Interface Residues | Interface area (Å2) | Salt bridges | Disulphide Bonds | Hydrogen Bonds |
|---|---|---|---|---|---|
| A B | 23 : 19 | 1749 : 1756 | 1 | - | 5 |
| A C | 20 : 22 | 1297 : 1324 | 2 | - | 3 |
| B C | 34 : 35 | 1689 : 1707 | 4 | - | 10 |

Salt bridges — Disulphide bonds — Hydrogen bonds — Non-bonded contacts

**Supplementary Figure S7:** PDBsum analysis of Mp1 or Mp58 and VPS52 multimer predictions. Part **(A)** provides the data for 1:1 stoichiometry with chain A representing Vps52 and B as Mp1. **(B)** shows 2:1 with VPS52 as chain A and Mp58 as chains B and C

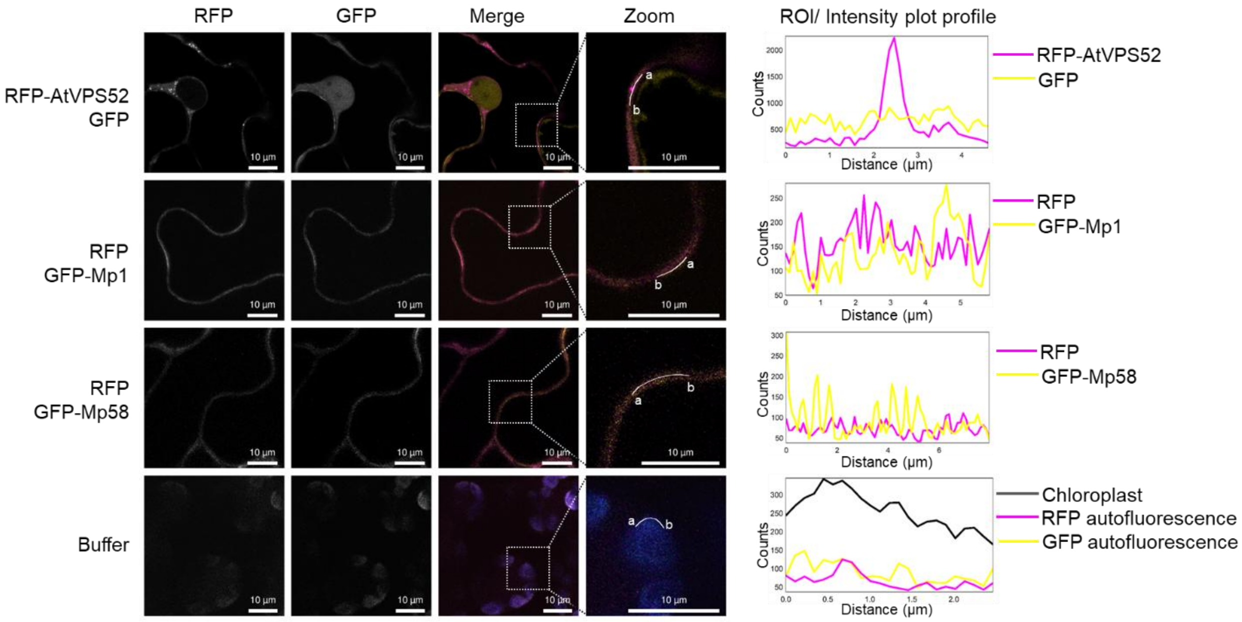


**Supplementary Figure S8:** Subcellular localisation of GFP-Mp1, GFP-Mp58 (in yellow), and RFP-AtVPS52 (in magenta) (controls for Fig. 3D) Proteins were transiently expressed *N. benthamiana* via agroinfiltration and localisation was observed with confocal microscopy. Magnification = 60X (water immersion lens), scale bar = 10 µm. Presented images are single plane images. The merged panel transect correspond to line intensity plot showing fluorescence distribution across the marked locus.

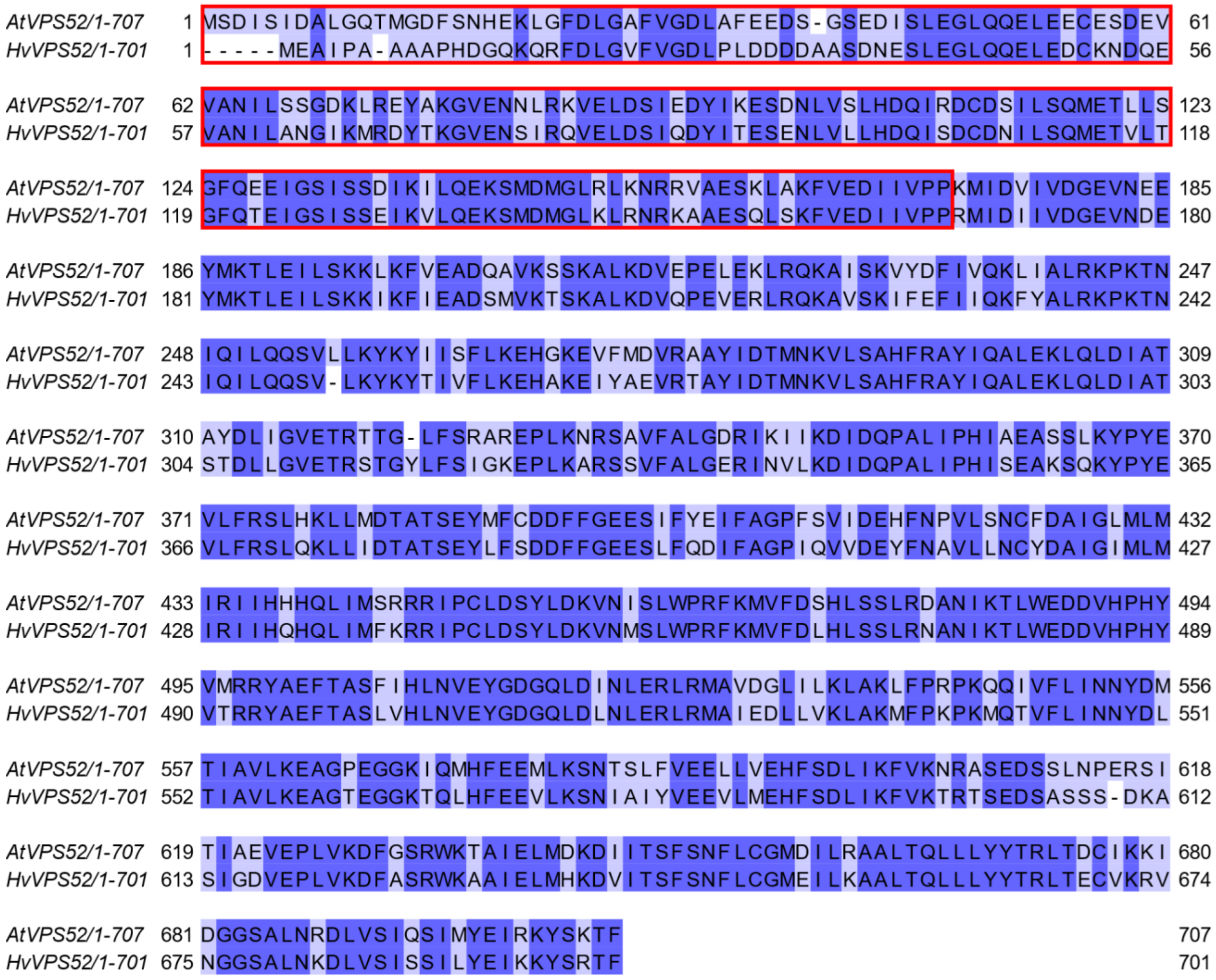


**Supplementary Figure S9:** Alignment of AtVPS52 and HvVPS52 amino acid sequences. The red box indicates N-terminal region swapped in VPS52 chimeras. Alignment created in Jalview

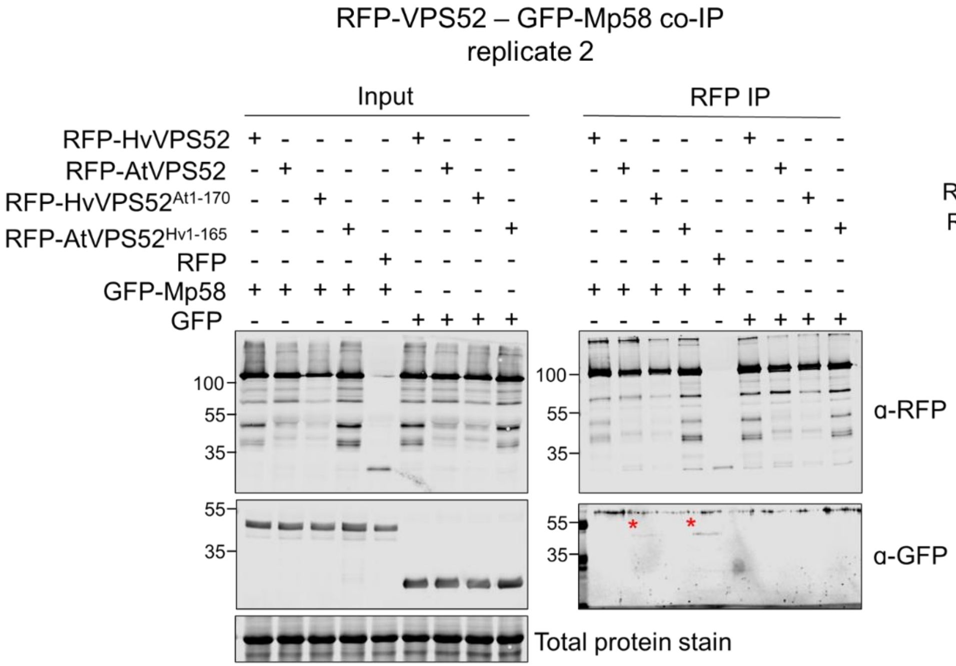


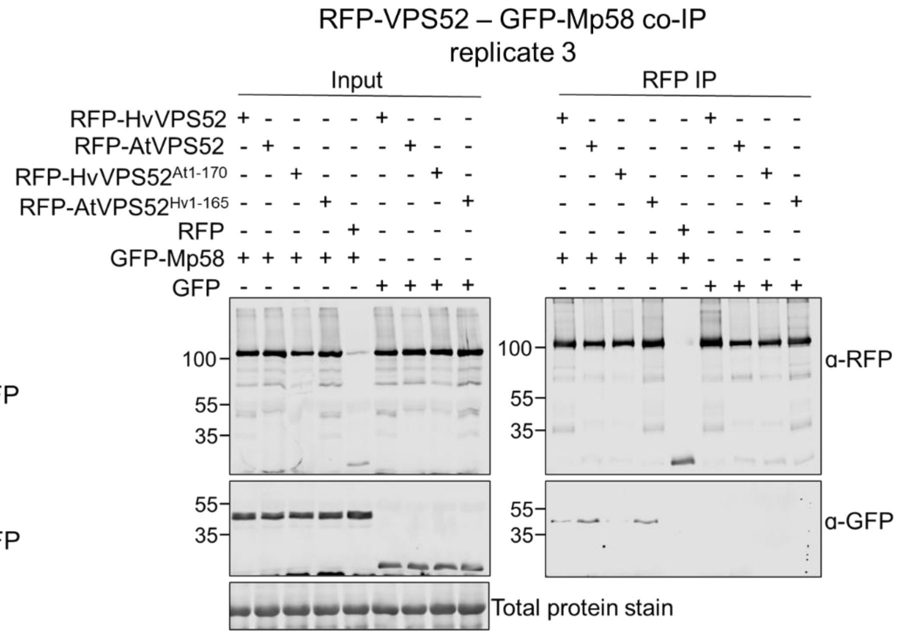


**Supplementary Figure S10:** Individual replicates for co-IP of RFP-tagged VPS52 variants with GFP-Mp58

GFP-Mp58 was co-expressed with RFP-HvVPS52, RFP-AtVPS52, or N-terminal chimeras in *N. benthamiana* via agroinfiltration. RFP-tagged proteins were pulled-down with RFP-trap and blotted against GFP. RFP and GFP were used as negative controls.

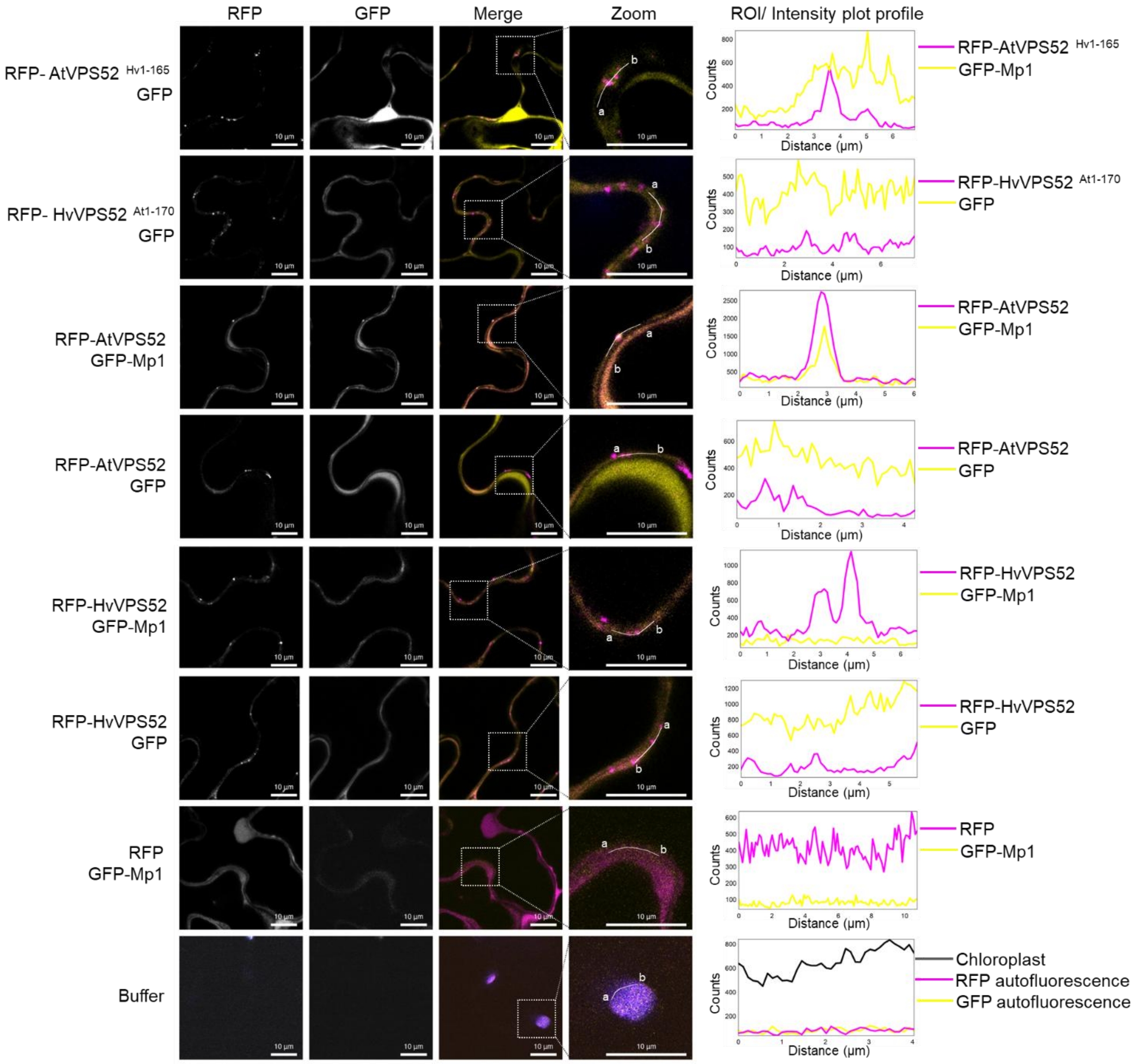


**Supplementary Figure S11:** Subcellular localisation of RFP-VPS52 variants (in magenta) and GFP-Mp1 (in yellow)

Proteins were transiently expressed in *N. benthamiana* via agroinfiltration, and subcellular localisation and localisation was observed with confocal microscopy. Magnification = 60X (water immersion lens), scale bar = 10 µm. Presented images are single plane images. The merged panel transect correspond to line intensity plot showing fluorescence distribution across the marked locus.

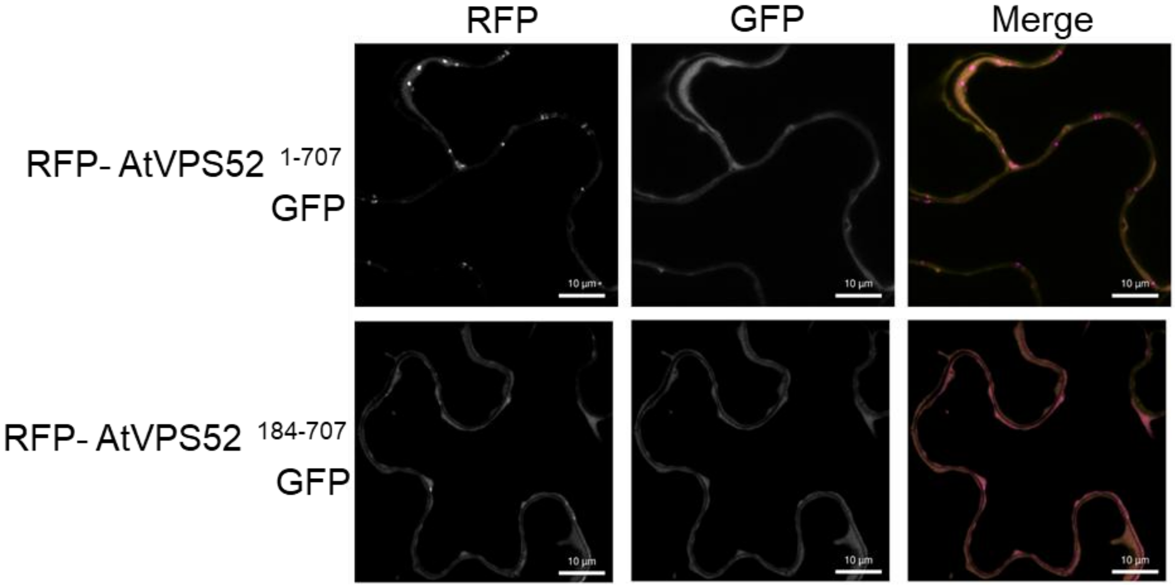


**Supplementary Figure S12:** Subcellular localisation of RFP-VPS52$^{184\text{-}707}$ (in magenta) compared to full length RFP-AtVPS52

Proteins were transiently expressed in *N. benthamiana* via agroinfiltration, and subcellular localisation and localisation was observed with confocal microscopy. Magnification = 60X (water immersion lens), scale bar = 10 µm. Presented images are single plane images. The merged panel transect correspond to line intensity plot showing fluorescence distribution across the marked locus.

## Uncropped western blots:

### Fig.1A uncropped

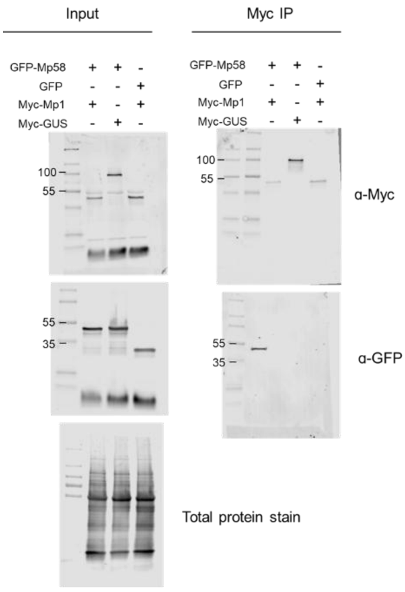


### Myc-Mp1 x GFP-Mp58 rep 2 (Fig S2)

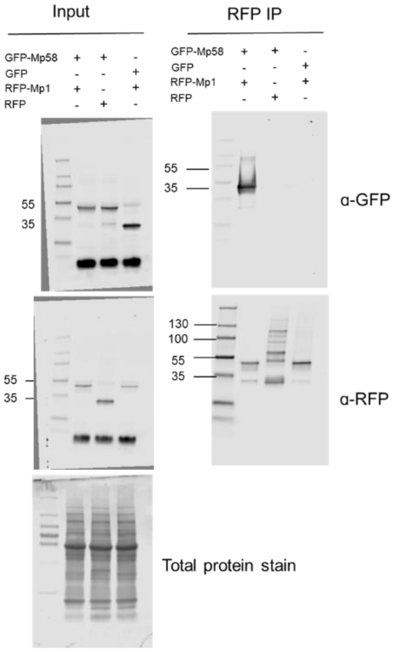


### Fig.1B uncropped

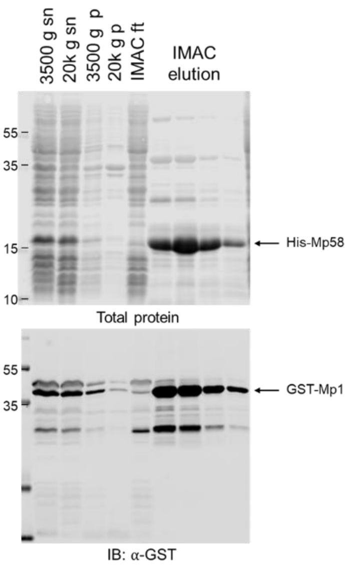


### Fig 2A uncropped (lanes 4-6 in main fig)

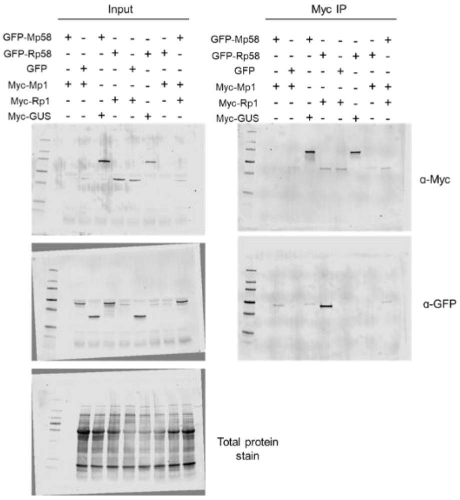


### Rp1-Rp58 co-IP rep 2

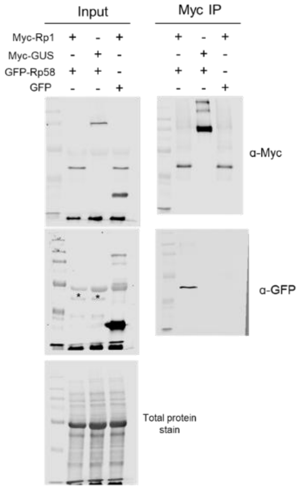

**Myc-Mp1 x GFP-Mp58/Rp58 Fig 2B uncropped**

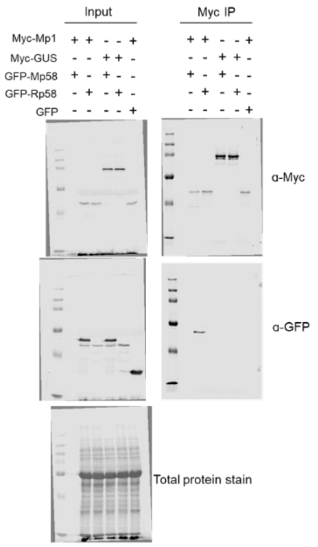


**Myc-Mp1 x GFP-Mp58/Rp58 rep2**

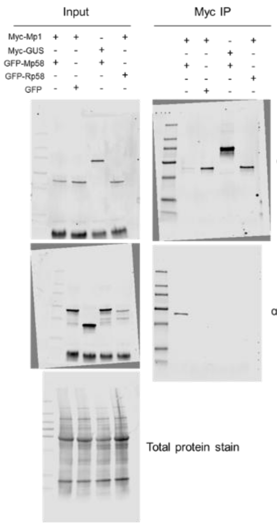


**Myc-Mp1 x GFP-Mp58/Rp58 rep3**

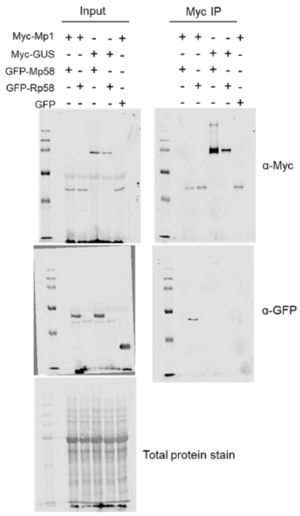


**Myc-Rp1 x GFP-Rp58/Mp58 Fig 2C uncropped**

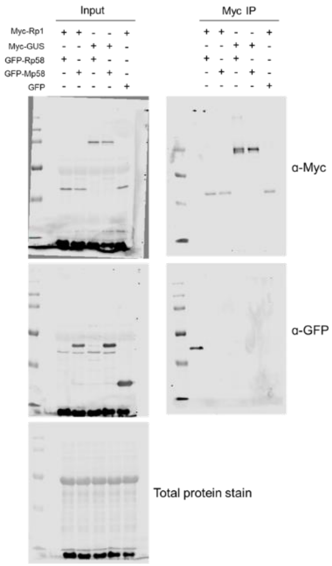


**Myc-Rp1 x GFP-Rp58/Mp58 rep2**

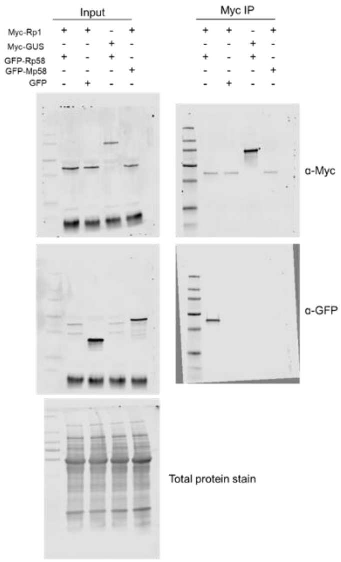


**Myc-Rp1 x GFP-Rp58/Mp58 rep3**

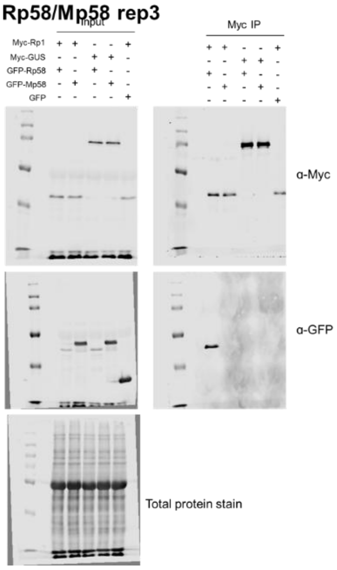

**RFP-AtVPS52 x GFP-Mp58 +/- myc-Mp1 Fig 3A uncropped blot**

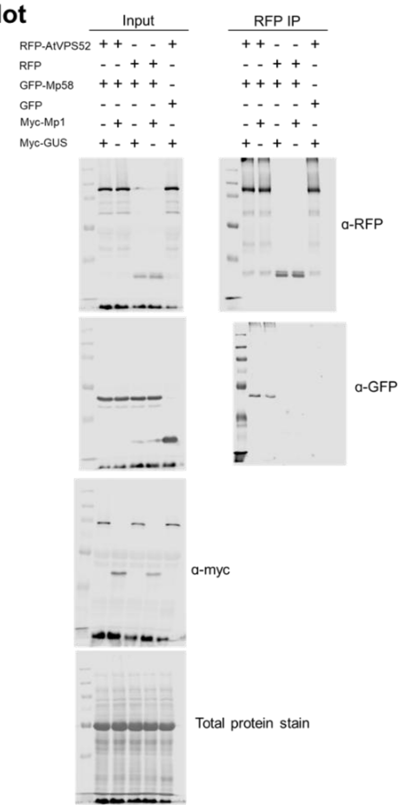


**RFP-AtVPS52 x GFP-Mp58 +/- myc-Mp1 rep 2**

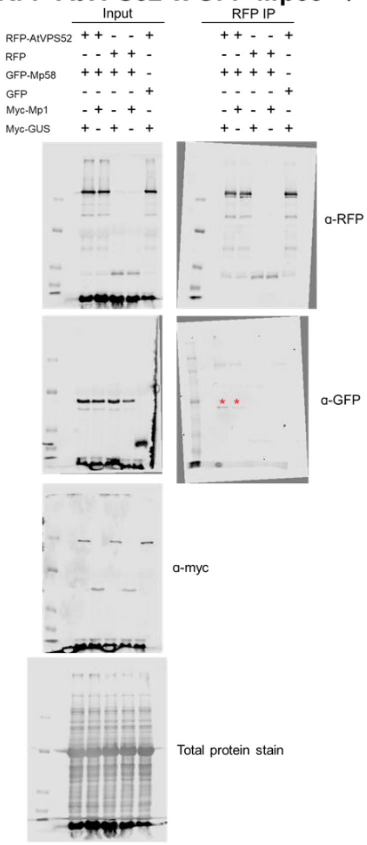


**RFP-AtVPS52 x GFP-Mp58 +/- myc-Mp1 Fig 3B uncropped blot**

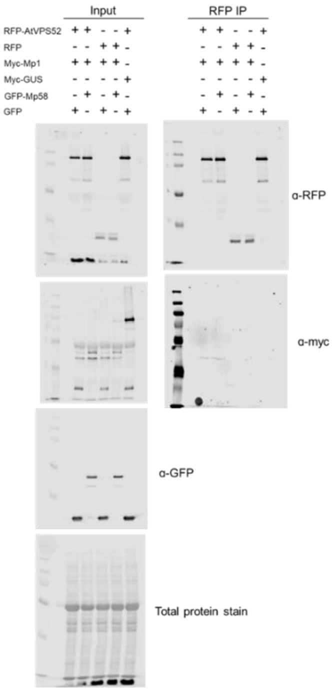


**RFP-AtVPS52 x myc-Mp1 +/- GFP-Mp58 rep 2**

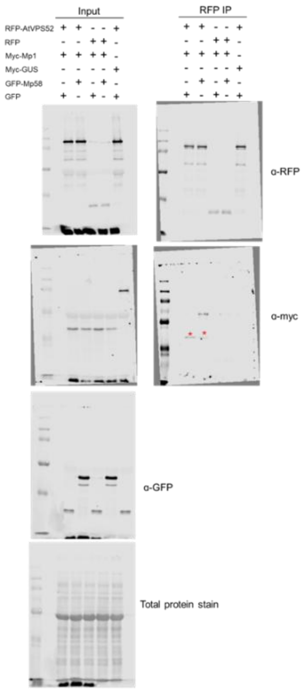

**Fig. 4B uncropped**

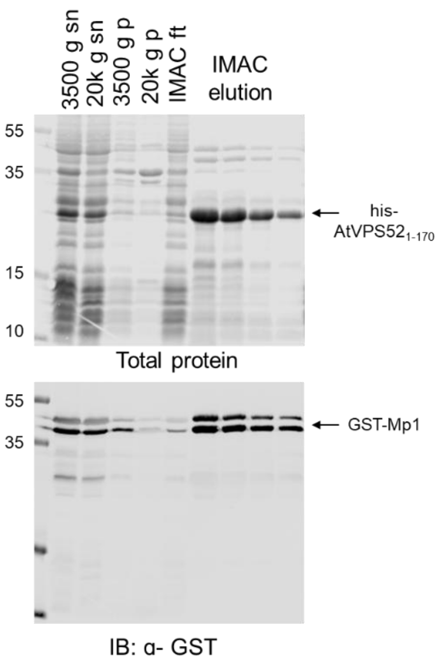


**Fig. 4C uncropped**

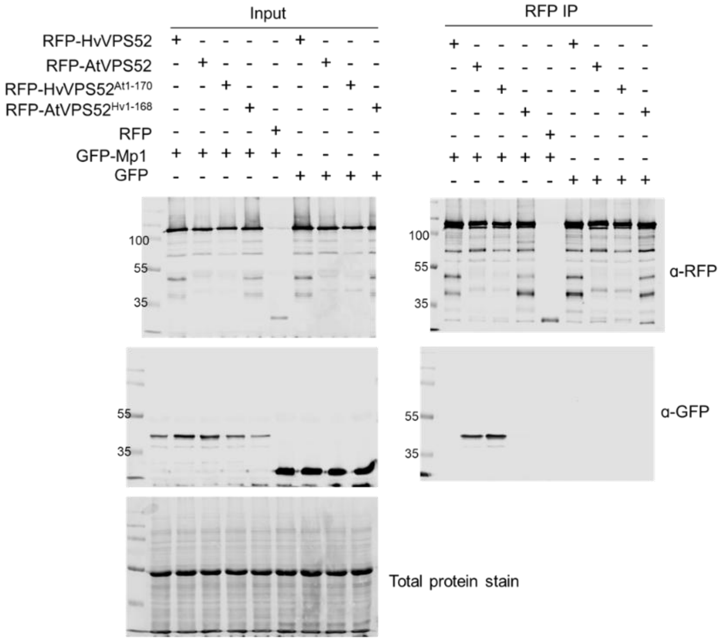


**RFP-VPS52s x GFP-Mp1 rep 2**

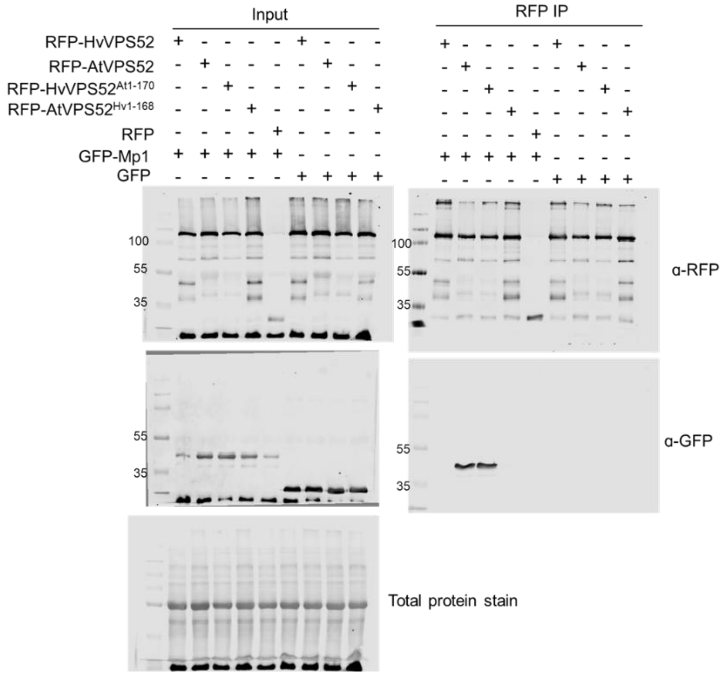


**Supplementary Figure 13:** Uncropped western blots for Figs 1, 2, 3, 4 including additional replicates.

Uncropped western blots and individual replicates for co-immunoprecipitation after Agroinfiltration (samples collected 3 days post infiltration) and purification of protein complexes expressed in *E. coli*.
